# *In vitro* and *in vivo* characterization of oridonin analogs as anti-inflammatory agents that regulate the NF-κB and NLRP3 inflammasome axis

**DOI:** 10.3389/fphar.2025.1512740

**Published:** 2025-02-27

**Authors:** Huiping Ou, Zhanpan Wu, Jinhua Ning, Qiufeng Huang, Wancun Wang, Guochun Yang, Yingxun Zhou, Anguo Hou, Peng Li, Lingyun Chen, Wen Bin Jin

**Affiliations:** ^1^ Faculty of Chinese Materia Medica, Yunnan University of Chinese Medicine, Kunming, Yunnan, China; ^2^ School of Food and Drug, Shenzhen Polytechnic University, Shenzhen, Guangdong, China

**Keywords:** oridonin analogs, anti-inflammation, NLRP3 inflammasome, NF-κB signaling pathway, acute lung injury

## Abstract

**Introduction:**

A series of oridonin hybrids were synthesized and evaluated for anti-inflammatory potential, focusing on their ability to inhibit NO production in RAW264.7 cells and their therapeutic prospects for NLRP3-driven disorders.

**Methods:**

Anti-inflammatory activity was assessed by measuring NO inhibition in LPS-stimulated RAW264.7 cells. The most active compound, 4c, was further analyzed using ELISA and WB to evaluate its effects on inflammatory proteins (p-NF-κB, p-IκB, NLRP3, IL-6, IL-1β, COX-2, iNOS). *In vivo* efficacy was tested in a murine acute lung injury model, with RT‒qPCR and WB used to assess inflammatory markers in lung tissues. Molecular docking predicted **4c**’s binding mode with NLRP3, while RNA-seq and RT‒qPCR identified differentially expressed genes.

**Results:**

Compound **4c** significantly inhibited NO production and suppressed key inflammatory proteins in vitro. In vivo, it alleviated acute lung injury, reduced IL-6 and TNF-α mRNA levels, and inhibited NLRP3, p-NF-κB, and IL-6 protein expression. Docking suggested covalent binding to NLRP3. RNA-seq revealed **4c** upregulated Trdc, Stfa2, and Gsta2 while downregulating Spib, Csf2, and Nr4a1.

**Discussion:**

Compound **4c** demonstrates potent anti-inflammatory effects via NLRP3 pathway inhibition and modulation of inflammatory genes. These findings highlight oridonin hybrids, particularly **4c**, as promising candidates for NLRP3-driven inflammatory disorders, warranting further investigation.

## Graphical Abstract

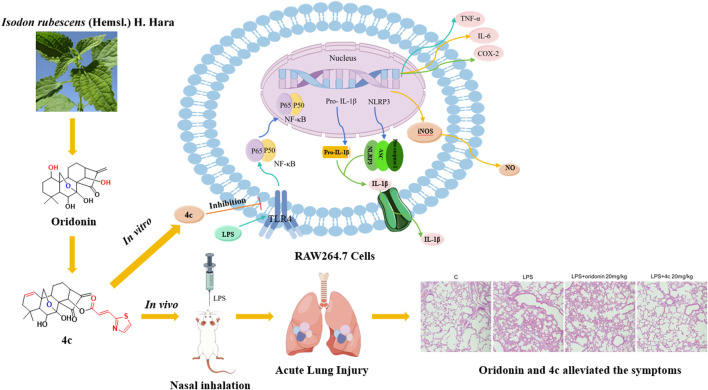

## 1 Introduction

Inflammation is a double-edged sword that underlies a wide variety of physiological and pathological processes. It is an adaptive response to noxious stimuli and multiple diseases, including tissue injury (Kumar, 2020), autoimmune diseases (Zanatta et al., 2019), type 2 diabetes (Halim and Halim, 2019), cardiovascular diseases (Soysal et al., 2020) and depression (Beurel et al., 2020). Mechanistically, the inflammatory response involves the delivery of immune cells to the site of infection or injury, followed by the activation of immune system components, such as Toll-like receptors (TLRs) and nucleotide-binding oligomerization-domain protein (NOD)-like receptor (NLR)3, thereby assisting in restoring damaged tissues (Li et al., 2020). Once infection has been recognized, a variety of inflammatory mediators, including chemokines, cytokines, vasoactive amines, eicosanoids and products of proteolytic cascades, are produced (Medzhitov, 2008; Barton, 2008). However, excessive activation of adaptive immunity may also trigger the overproduction of proinflammatory cytokines, resulting in pyroptotic cell death (Spel and Martinon, 2020). NLR family pyrin domain containing 3 (NLRP3) is a member of the NLR subfamily. The NLRP3 inflammasome is a multiprotein complex composed of the innate immune sensor NLRP3, an adaptor protein (apoptosis-associated speck-like protein containing CARD, ASC), and caspase-1. Upon assembly of the NLRP3 inflammasome, caspase-1 is activated, which promotes the cleavage of pro-IL-1*β* and pro-IL-18 to produce mature and functional IL-1β and IL-18, which play central roles in innate immunity and inflammation (Davis et al., 2011; Jo et al., 2016). Thus, targeting the NLRP3 inflammasome could be a promising strategy for anti-inflammatory drug discovery (Li et al., 2023).

Historically, many first-line chemotherapy drugs have been derived from phytochemicals (Harvey et al., 2015). Oridonin, a bioactive ent-kaurane diterpenoid isolated from *Rabdosia rubescens*, a commercially available over-the-counter (OTC) herbal medicine (Fujita et al., 1976), has attracted great attention for its considerable anti-inflammatory activity due to its ability to regulate NF-κB activation to suppress the release of proinflammatory cytokines. Recently, oridonin was reported to be a covalent inhibitor that targets the NLRP3 inflammasome. Oridonin was found to covalently bind to Cys279 located in the NACHT domain to disrupt the interaction between NLRP3 and NEK7, which results in failure of the assembly and activation of the NLRP3 inflammasome (He et al., 2018). Further in-depth studies of oridonin analogs that target the NLRP3 inflammasome have been reported (Pang et al., 2023; He et al., 2024). However, a structure‒activity relationship (SAR) study of oridonin is still lacking. Therefore, the oridonin scaffold could be worthy of further development for the treatment of NLRP3-driven disorders.

In this study, we synthesized a collection of novel oridonin hybrids and investigated their anti-inflammatory activities both *in vitro* and *in vivo* in an acute lung injury (ALI) animal model. The SAR study indicated that nearly all the synthetic oridonin derivatives exhibited remarkable anti-inflammatory activity, especially compound **4c**, which exhibited 17-fold greater anti-inflammatory activity than oridonin, demonstrating that deletion of the OH group at C-1 was preferable. Moreover, the 7,20-epoxy ent-kaurane diterpenoid scaffold generated by the diethylaminosulfur trifluoride (DAST) rearrangement of oridonin displayed lower anti-inflammatory activity. Moreover, we explored the inflammation-associated signaling pathways regulated by compound **4c**. Overall, we identified an oridonin derivative, **4c**, that targets the NF-κB and NLRP3 axis and exhibits striking anti-inflammatory activity both *in vitro* and *in vivo*.

## 2 Materials and methods

### 2.1 Chemistry

All commercial reagents and solvents used were provided by Aladdin Holdings. Group Co., Ltd. (Shanghai, China) and were used directly without further purification unless otherwise stated. The reactions were monitored by thin layer chromatography (TLC) and visualized via ultraviolet (UV) light at a wavelength of 254 nm. Chromatographic purifications were performed with silica gel (160–200 mesh) and gradient mixtures of petroleum ether and ethyl acetate as the eluent. ^1^H nuclear magnetic resonance (NMR) and ^13^C NMR spectra were measured on a Bruker Avance spectrometer. High-resolution mass spectrometry (HRMS) was performed on an Agilent 6,545 instrument in quadrupole time-of-flight (Q-TOF) mode. All the tested compounds were >95% pure according to high-performance liquid chromatography (HPLC) analysis (Agilent 1,220, Germany). Intermediate compounds **4** and **5** were synthesized according to previously published methods (Yao et al., 2020; Luo et al., 2018). Compounds **4a**, **4e**, **5a**, and **5f** have been reported previously (Ning et al., 2024). The synthetic procedures for the preparation of oridonin hybrids and the spectrum of target compounds could be found in supplementary materials.

### 2.2 Biological evaluation

#### 2.2.1 Cell culture

RAW264.7 cells, provided by Servicebio (Wuhan, China), were cultured in Dulbecco’s modified Eagle medium (Gibco 11965092) supplemented with 1% (v/v) penicillin (100 U/mL), streptomycin (100 μg/mL) and 10% fetal bovine serum (FBS; Gibco, 10099141C), followed by incubation at 37°C in a 5% CO_2_ incubator.

#### 2.2.2 CCK-8 assay

A Cell Counting Kit-8 (NCM Biotech C6005, China) was used to assess cell viability. Briefly, RAW 264.7 cells were cultured in 96-well culture plates at a density of 2 × 10^4^ cells/well and incubated for 24 h before removal of the cell culture medium. Afterward, the cells were pretreated with 1 μg/mL lipopolysaccharide (LPS) (Beyotime S1732, China) for 1 h and then coincubated with the oridonin derivatives for 24 h. A mixture of 10 μL of CCK-8 reagent with 90 μL of cell culture medium was added to each well for incubation at 37°C for 50 min before the absorbance of the solution was measured at 450 nm by using a BioTek Synergy HTX Multi-Mode Reader.

#### 2.2.3 Nitric oxide release assays and ELISAs

The production of NO was determined with a NO assay kit (Beyotime S0021M, China) according to the manufacturer’s instructions. Briefly, the cell culture supernatant was mixed with Griess Ⅰ and Griess II in sequence and then incubated at room temperature for 10 min as described in our previous report (Zhang et al., 2024; Li et al., 2024). The absorbance was measured at 540 nm with a BioTek Synergy HTX Multi-Mode Reader. ELISAs for IL-1β and IL-6 were performed via ELISA kits (Boster EK0394, EK0411, China) according to the manufacturer’s instructions.

#### 2.2.4 Western blot analysis

The total protein was extracted from the cells and lung tissues with RIPA lysis buffer (Beyotime P0013, China). The proteins were separated by SDS‒PAGE and transferred to a PVDF membrane before 1 h of blocking with 5% skim milk at room temperature. The membranes were further incubated overnight at 4°C with primary antibodies (Cell Signaling Technology: *β*-actin, 8H10D10; COX-2, D5H5; NLRP3, D4D8T; IL-6, D5W4V; P-IκBα, 14D4; IκBα, L35A5; P-NF-κB, 93H1; NF-κB, D14E12; and GAPDH: D16H11) at a dilution of 1:1,000. Afterward, the membranes were incubated with secondary antibodies (Cell Signaling Technology: anti-mouse: 7,076; and anti-rabbit: 7,074) at a 1:1,000 dilution for 1 h at room temperature. The membranes were subsequently exposed to a ChemiDoc imaging system (Bio-Rad) after treatment with enhanced chemiluminescence (ECL) substrate.

#### 2.2.5 Animal experiments

BALB/c male mice aged 6–8 weeks and weighing 18–22 g were purchased from Zhuhai Bestest Biotech Co., Ltd. (certificate SCXK20200051; Zhuhai, China). The animals were housed in an environment with constant room temperature on a 12/12 h light−dark cycle and had free access to food and water. The mice were randomly divided into four groups: control (PBS with 10% DMSO), LPS (1.25 mg/kg in PBS), LPS (1.25 mg/kg in PBS) + oridonin (20 mg/kg in PBS with 10% DMSO), and LPS (1.25 mg/kg in PBS) + **4c** (20 mg/kg in PBS with 10% DMSO). Oridonin and **4c** were injected intraperitoneally, followed by LPS infusion into the nasal cavity 1 h later. The mice were euthanized by carbon dioxide (100%) asphyxiation 24 h after LPS administration, and then lung tissue samples were collected for analysis. Histological analysis of the lung tissues was performed after the tissues were immersed in 4% paraformaldehyde, embedded in paraffin, cut into 4 μm sections, and stained with hematoxylin‒eosin (HE) before examination with a microscope (Nikon, Japan).

#### 2.2.6 Immunohistochemistry staining

The paraffin slides were dewaxed and rehydrated. An antigen retrieval protocol using heat was used to unmask the antigens (30 min in citrate buffer 0.01 M, pH 6.0), then block the slides with serum blocking reagent. The slides were incubated with LCA (CD45) primary antibody (60287-1-lg, Proteintech, United States) overnight at 4°C. After washing with TBST for three times, the slides were incubated with the secondary antibody. Washing slides with TBST for three times then adding DAB Chromogen Solution to cover the entire tissue section and incubate for 10 min. Slides were lightly counterstained with hematoxylin to reveal nuclei, examined and photographed with a microscope.

#### 2.2.7 qPCR and RNA-seq assays

Total RNA from the cells and lung tissues was extracted with an RNeasy^®^ RNA extraction kit (Solarbio R1200, China) and dissolved in enzyme-free water. cDNA was synthesized with PrimeScript RT Master Mix (Takara RR036A, Japan) on a Biometra TAdvanced 96 SG (Analytik Jena, Jena, Germany). The total cDNA was used as the starting material for qPCR with GOTaq qPCR Master Mix (Promega A6002, United States) on a StepOnePlus Real-Time PCR system (Thermo Fisher, United States) according to the manufacturer’s instructions. The relative expression of the target genes was calculated via the 2^−ΔΔCT^ method, with GAPDH used as a control. The mouse cDNA primer sequences for qPCR are listed in the supplementary materials (Supplementary Table S1).

RAW 264.7 cells were seeded in 3 cm dishes, treated with 1 μg/mL LPS for 1 h and then coincubated with oridonin derivatives for 24 h before RNA extraction. The samples were prepared in triplicate. Total RNA was extracted with TRIzol reagent (15596026, Invitrogen) according to the manufacturer’s instructions. Preparation of the RNA library and transcriptome sequencing were conducted by Novogene Co., Ltd. (Beijing, China). Genes with adjusted p values <0.05 and |log2 (fold change)| values > 1 were considered to be significantly differentially expressed.

#### 2.2.8 Molecular docking studies

AutoDockFR (ADFR) software was used for molecular docking. The 3D structure of compound 4c was generated in ChemDraw 22.0, followed by MM2 energy optimization. The X-ray crystal structure of the NLRP3 protein served as the receptor after the elimination of the unnecessary water molecules and small ligands bound to the protein. The Ori-NLRP3 protein interaction sites were defined according to a published paper (He et al., 2018). The binding results were visualized with PyMOL version 2.5.2 software.

#### 2.2.9 Statistical analysis

GraphPad Prism (Version 9.5.1) was used for statistical analysis. The results are expressed as the means ± SDs of data from at least 3 independent experiments. Student’s t-test or one-way ANOVA was used to determine the statistical significance of differences between two or more groups. A p value < 0.05 was considered to indicate statistical significance.

## 3 Results

### 3.1 Chemistry

**SCHEME 1:**
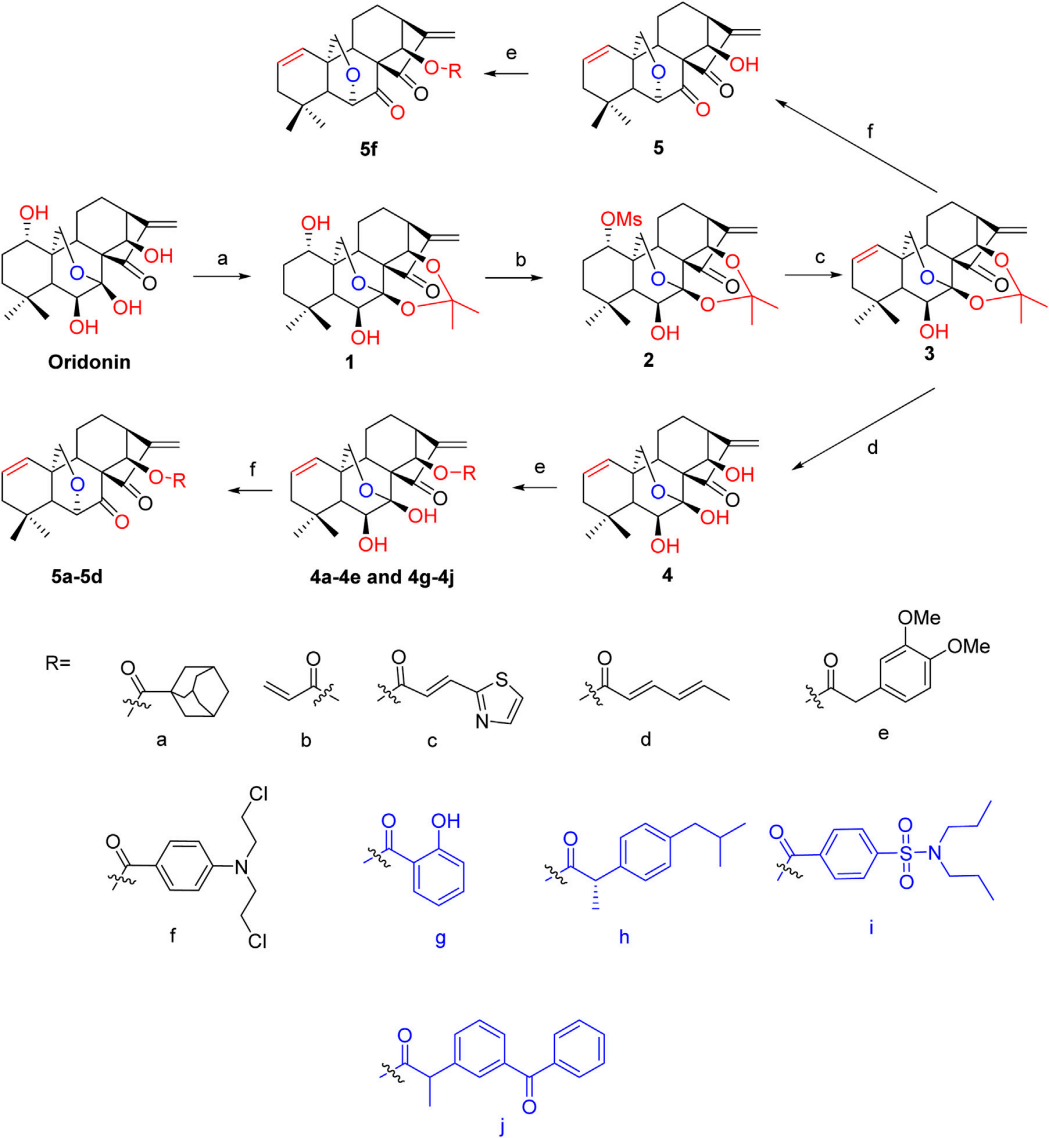
Synthesis of oridonin derivatives **4a-4e, 4g-4j, 5a-5d** and **5f** Reagents and conditions: **(A)** 2,2-dimethoxypropane, TsOH, CHCl_3_, reflux, 4 h; **(B)** MsCl, TEA, DCM, 0°C to rt; **(C)** LiBr, Li_2_CO_3_, DMF, 120°C, 4 h; **(D)** 10% HCl/THF (1:1), rt, 2 h; **(E)** corresponding acids, DMAP, EDCI, HOBt, DCM, rt, overnight; **(F)** DAST, DCM, −78°C for 20 min, N_2_ atmosphere, rt for 2 h.

By selectively deleting or blocking the OH groups of the oridonin scaffold, as well as DAST rearrangements or combination with FDA-approved nonsteroidal anti-inflammatory drugs (NSAIDs), 14 oridonin hybrids were synthesized *de novo*. On the basis of previously published methods, the synthetic procedures are depicted in Scheme 1 below (Yao et al., 2020; Ding et al., 2013; Xu et al., 2017). Initially, the selective protection of oridonin with 2,2-dimethoxypropane catalyzed by TsOH afforded compound 1, which upon 1-OH group protection via reaction with MsCl yielded 2, followed by an elimination reaction driven by LiBr and Li_2_CO_3_ to afford compound 3. Subsequently, acetonide removal from **3** catalyzed by 10% HCl led to key intermediate 4, which further underwent coupling reactions with various well-known acids, such as FDA-approved NSAIDs (salicylate, ibuprofen, ketoprofen, and probenecid) and adamantane-1-carboxylic acid at the C-14 hydroxy position, to afford oridonin derivatives **4a-4e** and **4g-4j**. Compounds **5a-5d** were further afforded via the DAST-induced rearrangement of compounds **4a-4d**. Notably, intermediate **3** directly underwent selective deprotection and rearrangement to give 6,20-epoxy-14-OH ent-kaurane diterpenoid **5** in just one step. Further conjugation of 5 with the nitrogen mustard gave hybrid **5f**. All of these products were easily purified by flash chromatography.

### 3.2 Biological evaluation

#### 3.2.1 Griess assay for NO release assessment and cytotoxicity determination

**FIGURE 1:**
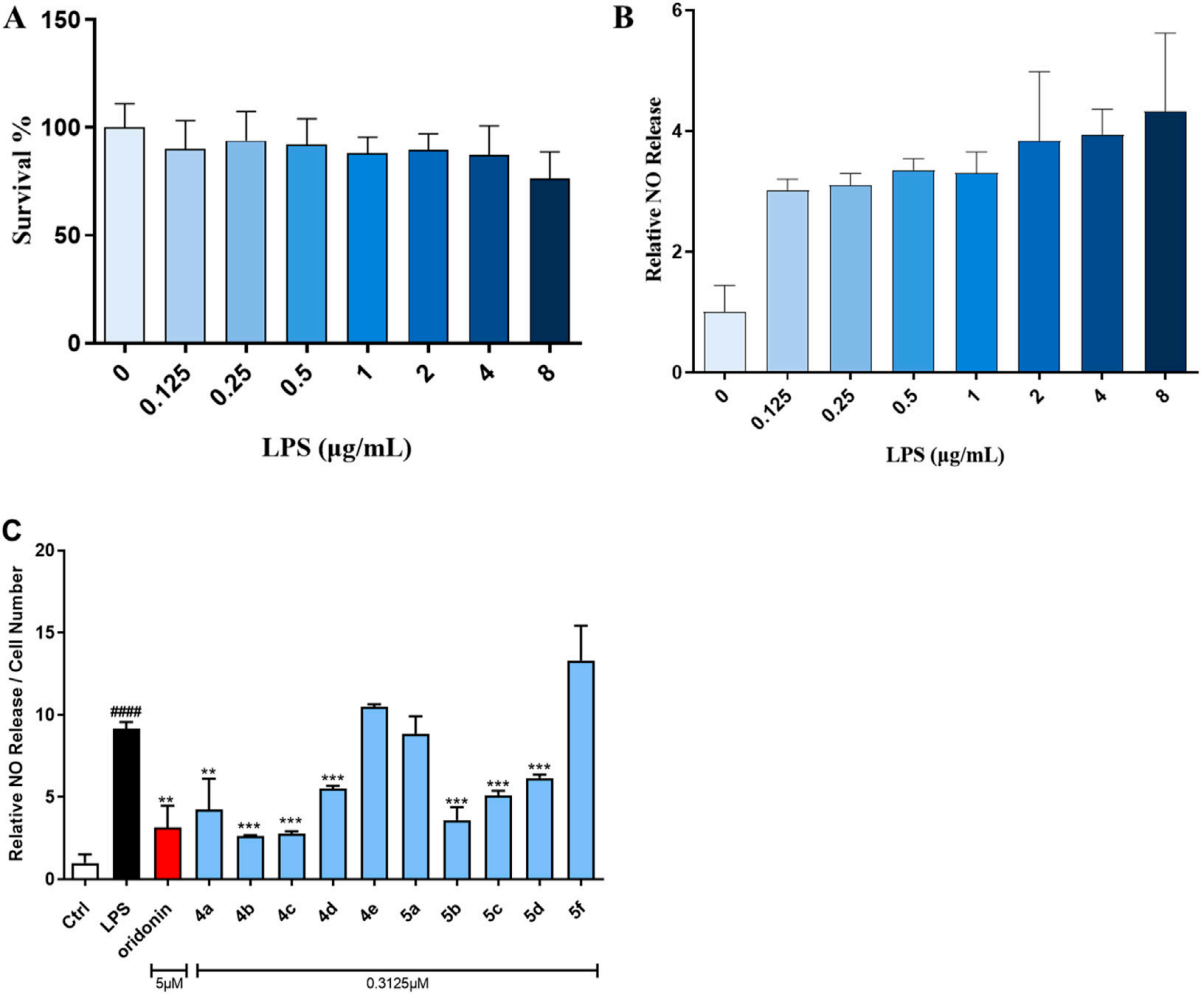
Oridonin derivatives inhibited the LPS-induced release of NO from RAW264.7 cells. **(A)**, CCK-8 assay with RAW264.7 cells treated with various concentrations of LPS. **(B)**, NO release assay with RAW264.7 cells treated with various concentrations of LPS. **(C)**, NO release assay with RAW264.7 cells treated with oridonin and its derivatives. The cells were stimulated with 1 μg/mL LPS for 1 h, cocultured with the oridonin derivatives for 24 h, and subjected to a NO release assay. All the results are expressed as the average value ± SD of three independent experiments. # indicates that the difference between the LPS and control groups is significant; and * indicates that the difference between the oridonin derivative and LPS groups is significant. Student’s t-test was used to calculate the significance, #### and ****, p < 0.0001; ### and ***, p < 0.001; ## and **, p < 0.01; # and *, p < 0.05.

NO is strongly associated with the pathogenesis of inflammatory diseases. The Griess assay for NO production was considered the gold standard for screening oridonin analogs for the treatment of inflammatory-associated disorders. Initially, the appropriate concentration of LPS to induce inflammation in the RAW264.7 macrophage line was investigated. As shown in Figures 1A, B, LPS at concentrations ranging from 0.125 to 8 μg/mL showed no toxicity to RAW264.7 cells and could stimulate NO production in these cells. Hence, 1 μg/mL LPS was utilized to evaluate the ability of the oridonin derivatives to inhibit NO release. In preliminary experiments, we reported that the highest concentrations of oridonin and its derivatives with no severe cytotoxicity were 5 μM and 0.3125 μM, respectively. Therefore, the initial treatment concentrations of oridonin and its analogs were set to these two concentrations. Notably, treatment of RAW264.7 cells with oridonin and its derivatives significantly suppressed the release of NO induced by LPS at the abovementioned concentrations (Figure 1C). In summary, these oridonin derivatives significantly inhibited LPS-induced NO release from RAW264.7 cells.

#### 3.2.2 Oridonin analogs with a 7,20-epoxy ent-kaurane diterpenoid scaffold exhibited increased anti-inflammatory activity

**FIGURE 2:**
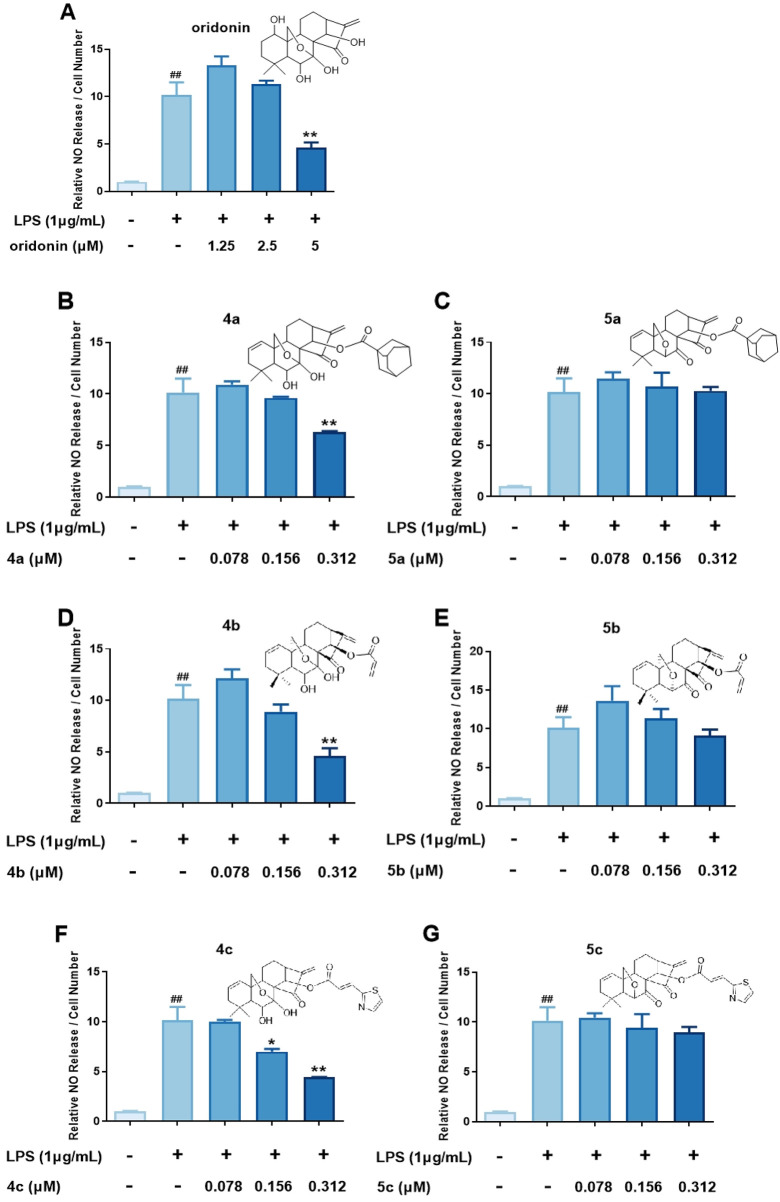
Determination of the IC_50_ values of the oridonin analogs. **(A–G)**, NO release assay with **4a-c**, **5a-c** and oridonin. The cells were stimulated with 1 μg/mL LPS for 1 h, cocultured with oridonin derivatives for 24 h, and then subjected to a NO release assay. All the results are expressed as the average value ± SD of three independent experiments. # indicates that the difference between the LPS and control groups is significant; and * indicates that the difference between the oridonin derivative and LPS groups is significant. Student’s t-test was used to calculate the significance, ### and ***, p < 0.001; ## and **, p < 0.01; # and *, p < 0.05.

On the basis of the initial anti-inflammatory activity screening results (Figure 1C), three pairs of oridonin derivatives, **4a** and **5a**, **4b** and **5b**, and **4c** and **5c**, which had high structural similarity and promising anti-inflammatory activity, were further investigated for IC_50_ determination. The IC_50_ values of the oridonin derivatives for NO inhibition are shown in Table 1. As shown in Figures 2A–G, oridonin derivatives **4a**, **4b** and **4c** with 7,20-epoxy ent-kaurane diterpenoid scaffolds had greater inhibitory effects on NO release than did **5a**, **5b** and **5c** with 6,20-epoxy ent-kaurane diterpenoid scaffolds, suggesting that DAST rearrangement of the scaffold did not significantly improve the anti-inflammatory activity of the compound in RAW246.7 cells. Compound **4c**, with an IC_50_ of 0.28 ± 0.01 μM, displayed the highest anti-inflammatory activity, with approximately 17-fold greater anti-inflammatory activity than oridonin in the NO release assay.

**TABLE 1:** Summary of the IC_50_ values of compounds **4a-c**, **5a-c** and oridonin for the inhibition of NO production.

Compound	IC_50_ (μM)	7,20-Epoxy ent-kaurane diterpenoid scaffold	6,20-Epoxy ent-kaurane diterpenoid scaffold
Oridonin	4.85 ± 0.15		
4a	0.37 ± 0.01	√	
5a	1.03 ± 0.15		√
4b	0.30 ± 0.02	√	
5b	4.21 ± 3.99		√
4c	0.28 ± 0.01	√	
5c	0.58 ± 0.16		√

#### 3.2.3 Oridonin hybrids exhibited decreased anti-inflammatory activity

**FIGURE 3:**
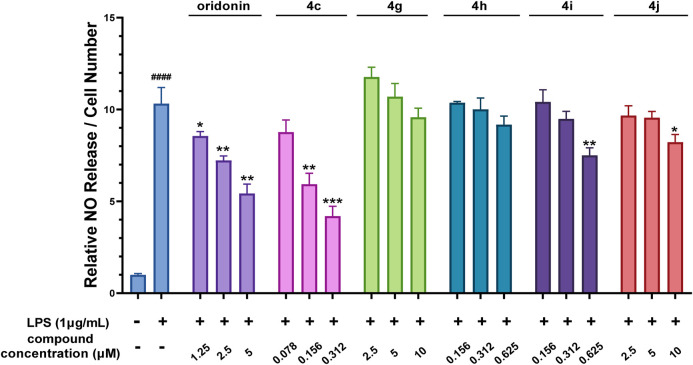
The oridonin hybrids had weak anti-inflammatory activity. NO release assay with the oridonin hybrids. The cells were stimulated with 1 μg/mL LPS for 1 h, cocultured with oridonin derivatives for 24 h, and then subjected to a NO release assay. All the results are expressed as the average value ± SD of three independent experiments. # indicates that the difference between the LPS and control groups is significant; and * indicates that the difference between the oridonin derivative and LPS groups is significant. Student’s t-test was used to calculate the significance, #### and ****, p < 0.0001; ### and ***, p < 0.001; ## and **, p < 0.01; # and *, p < 0.05.

Oridonin hybrids conjugated with FDA-approved NSAIDs were then synthesized to evaluate if there was a synergistic effect after hybridization. NO release assays (Figure 3) revealed that, compared with oridonin derivative **4c**, compounds **4g**-**j** presented lower anti-inflammatory activity.

#### 3.2.4 Compounds 4b and 4c inhibited the secretion of IL-6 and IL-1β

**FIGURE 4:**
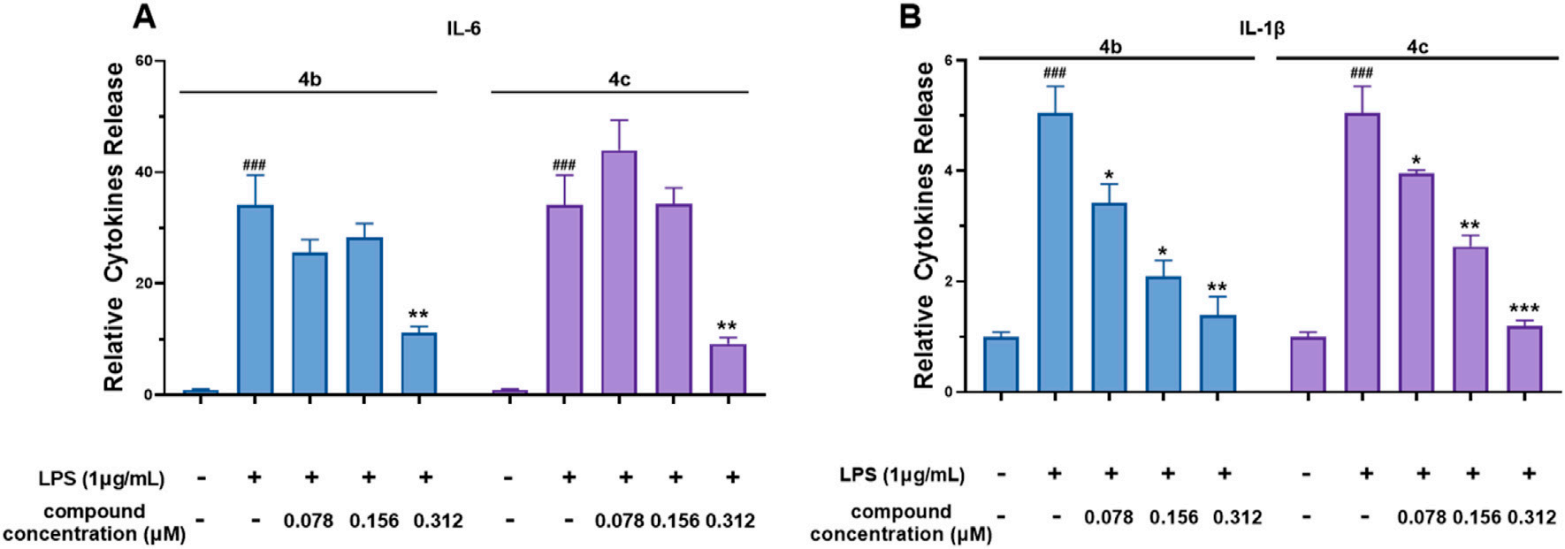
Compounds **4b** and **4c** inhibited the secretion of IL-6 and IL-1β. **(A)**, ELISA for IL-6. **(B)**, ELISA for IL-1β. RAW 264.7 cells were stimulated with 1 μg/mL LPS for 1 h, cocultured with **4b** and **4c** for 24 h, and then subjected to ELISA. All the results are expressed as the average value ± SD of three independent experiments. # indicates that the difference between the LPS and control groups is significant; and * indicates that the difference between the oridonin derivative and LPS groups is significant. Student’s t-test was used to calculate the significance, ### and ***, p < 0.001; ## and **, p < 0.01; # and *, p < 0.05.

Compounds **4b** and **4c**, which had the lowest IC_50_ values for NO production, were selected for further investigation of their inhibitory effects on IL-6 and IL-1β secretion from RAW 264.7 cells. The results shown in Figure 4A and **B** reveal that LPS dramatically induced IL-6 and IL-1β secretion. However, both **4b** and **4c** significantly inhibited the secretion of IL-6 and IL-1β in a dose-dependent manner. Accordingly, the IC_50_ values of compound **4b** against the secretion of IL-1β and IL-6 were 0.22 ± 0.02 μM and 0.23 ± 0.02 μM, respectively, whereas for compound **4c**, these values were 0.21 ± 0.02 μM and 0.21 ± 0.03 μM, respectively. These data indicate that the inhibitory effect of **4c** on the release of IL-6 and IL-1β was slightly greater than that of **4b**.

#### 3.2.5 Compounds 4b and 4c inhibited the LPS-stimulated expression of inflammatory genes at the mRNA level

**FIGURE 5:**
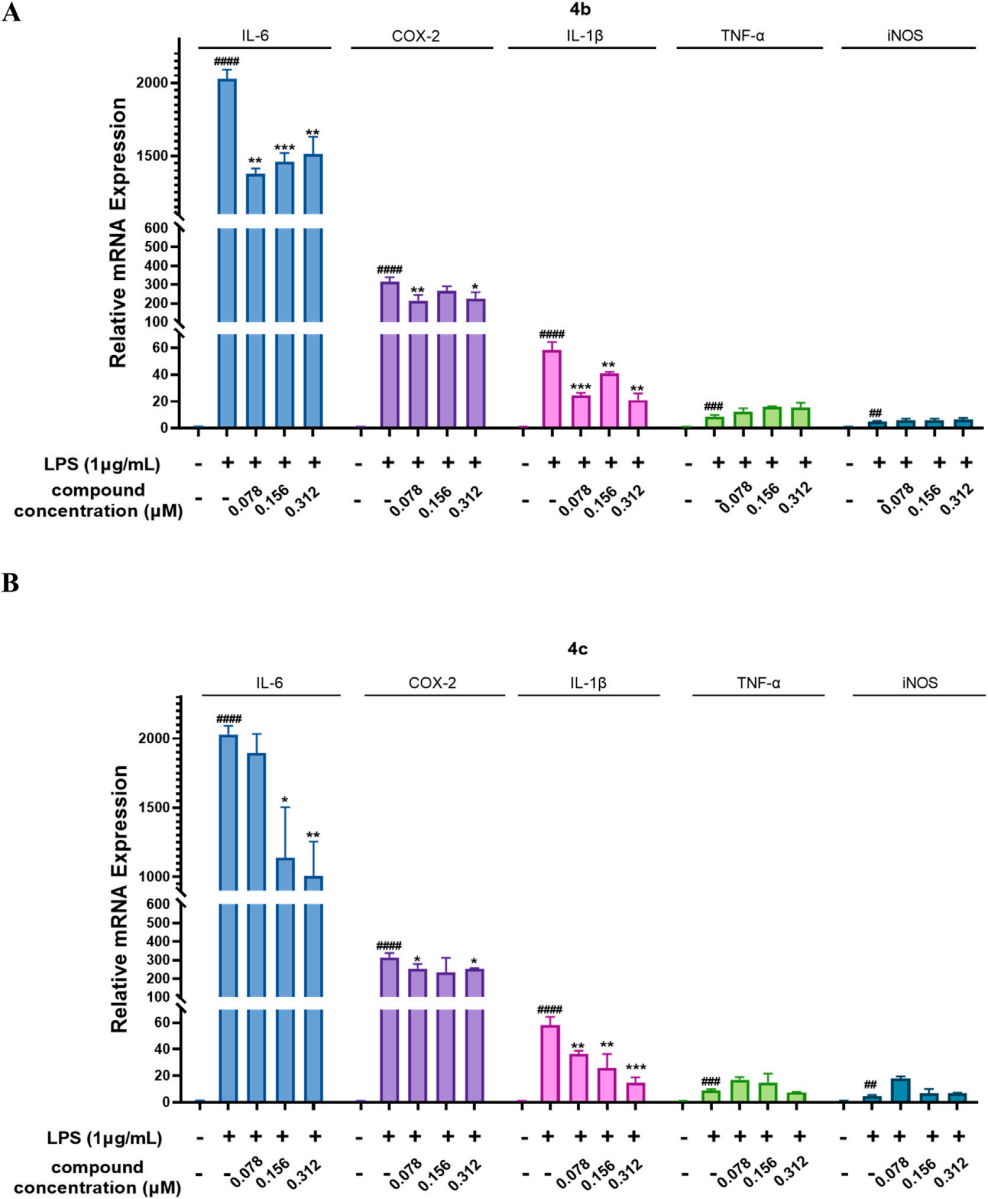
Compounds **4b** and **4c** inhibited the LPS-stimulated mRNA expression of inflammatory genes. **(A, B)**, qPCR assay of **4b** and **4c**. RAW264.7 cells were stimulated with 1 μg/mL LPS for 1 h, cocultured with **4b** or **4c** for 24 h, and then subjected to a qPCR assay. All the results are expressed as the average value ± SD of three independent experiments. # indicates that the difference between the LPS and control groups is significant; and * indicates that the difference between the oridonin derivative and LPS groups is significant. Student’s t-test was used to calculate the significance, #### and ****, p < 0.0001; ### and ***, p < 0.001; ## and **, p < 0.01; # and *, p < 0.05.

Next, we evaluated the inhibitory effects of **4b** and **4c** on inflammatory mediators at the mRNA level. As shown in Figures 5A, B, the qPCR results indicated that LPS enhanced the mRNA expression of the inflammatory genes IL-6, COX-2, IL-1β, TNF-α and iNOS in the RAW 264.7 cell line. Notably, compounds **4b** and **4c** inhibited the LPS-stimulated expression of the inflammatory genes IL-6, COX-2 and IL-1β. Moreover, **4c**, compared with **4b**, much more effectively inhibited the expression of these inflammatory genes. Unfortunately, neither **4b** nor **4c** inhibited the LPS-stimulated expression of the inflammatory genes iNOS and TNF-α. In summary, **4c** was more effective than **4b** in inhibiting the LPS-stimulated expression of the inflammatory genes IL-6, COX-2, and IL-1β, but not iNOS and TNF-α.

#### 3.2.6 Compounds 4b and 4c inhibited the expression of inflammation-related proteins

**FIGURE 6:**
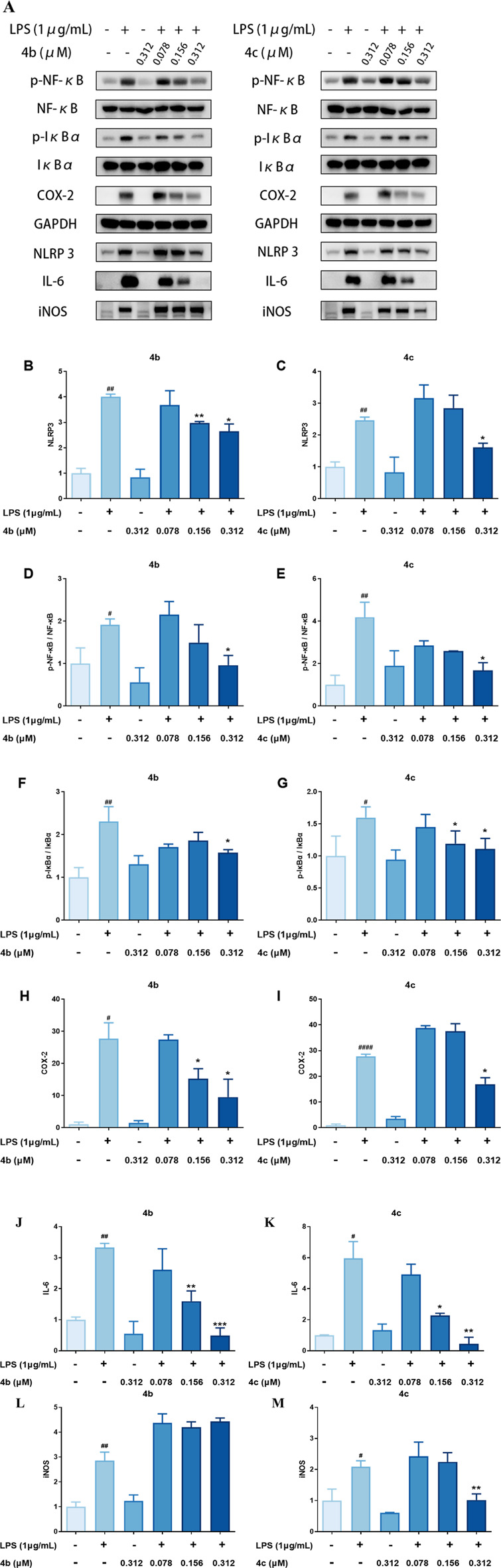
Compounds **4b** and **4c** inhibited the expression of inflammatory proteins. **(A)**, WB analysis of p-NF-κB, NF-κB, p-IκBα, IκBα, COX-2, NLRP3, IL-6 and iNOS. RAW264.7 cells were pretreated with LPS for 1 h, coincubated with **4b** and **4c** for 24 h, and then subjected to WB analysis. **(B–M)**, Quantification of the results of WB analysis of inflammatory proteins. All the results are expressed as the average value ± SD of three independent experiments. # indicates that the difference between the LPS and control groups is significant; and * indicates that the difference between the oridonin derivative and LPS groups is significant. Student’s t-test was used to calculate the significance, #### and ****, p < 0.0001; ### and ***, p < 0.001; ## and **, p < 0.01; # and *, p < 0.05.

Previous studies have indicated that oridonin is a covalent inhibitor of NLRP3. Therefore, we also measured the expression of inflammation-related proteins, including those involved in the NLRP3 inflammasome and the NF-κB pathway. As shown in Figures 6A–M, **4b** and **4c** regulated the NF-κB signaling pathway by inhibiting the LPS-induced expression of phosphorylated NF-κB and phosphorylated IκB, as well as their downstream target COX-2. Importantly, **4b** and **4c** also inhibited the NLRP3 inflammasome by inhibiting the expression of NLRP3 and its downstream effector IL-6. Interestingly, as shown in Figures 2D, F, compounds **4b** and **4c** significantly inhibited LPS-induced NO production. However, the results of the qPCR assay shown in Figure 5A and **B** indicated that **4b** and **4c** could not downregulate iNOS mRNA. However, WB analysis suggested that iNOS protein expression was significantly inhibited by **4c** but not by **4b**. In summary, **4c** was more effective than **4b** in inhibiting the LPS-induced expression of inflammatory proteins, including phosphorylated NF-κB, phosphorylated IκB, NLRP3, IL-6 and iNOS.

#### 3.2.7 Compound 4c alleviated the symptoms of ALI in mice

**FIGURE 7:**
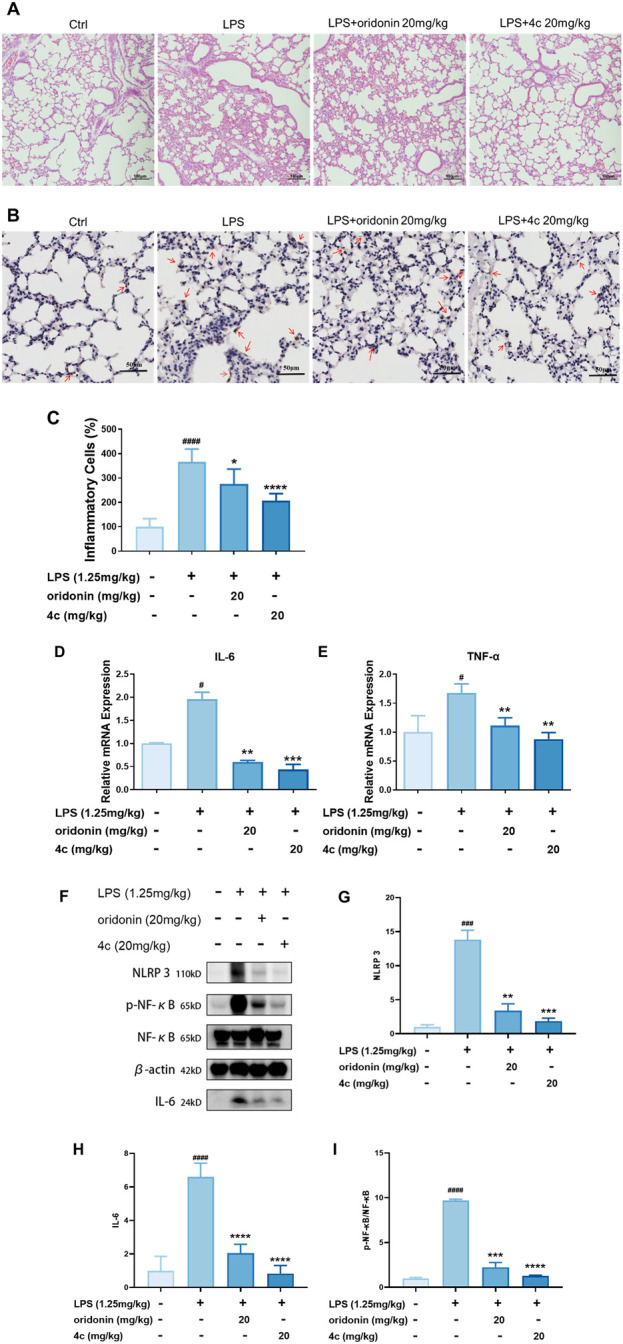
Compound **4c** alleviated the symptoms of acute lung injury in mice. **(A)**, Hematoxylin–eosin (HE) staining of lung tissue sections (magnification, ×100). ALI was established by nasal infusion of LPS. BALB/c mice were intraperitoneally injected with oridonin and 4c. **(B)**, Immunohistochemistry (IHC) staining of lung tissue sections with LCA (CD45) antibody and hematoxylin (magnification, ×300). **(C)**, Quantitation of the inflammatory cells (LCA positive staining cells) in the lung tissue sections. **(D‒E)**, RT‒qPCR assays of lung tissues. **(F)**, WB analysis of lung tissues. **(G‒I)**, Quantification of the WB results for NLRP3, p-NF-κB and IL-6. All the results are expressed as the average value ± SD of three independent experiments. # indicates that the difference between the LPS and control groups is significant; and * indicates that the difference between the oridonin derivative and LPS groups is significant. Student’s t-test is used to calculate the significance, ####and ****, p < 0.0001; ### and ***, p < 0.001; ## and **, p < 0.01; # and *, p < 0.05.

We verified that these oridonin derivatives, among which **4c** was the most promising, showed promising *in vitro* anti-inflammatory activity. Next, we established a mouse model of ALI to determine the *in vivo* anti-inflammatory activity of **4c**. As shown in Figure 7A, after nasal inhalation of LPS, the inflammatory cell infiltration in the lung tissue of the mice was examined. Notably, inflammatory infiltration was partially alleviated by oridonin (20 mg/kg) and fully alleviated by **4c** (20 mg/kg). Moreover, we performed IHC staining of the lung tissue with leukocyte common antigen (LCA, also known as CD45) antibody as a biomarker of inflammatory cells. Figures 7B,C indicated that the percentage of LCA positive inflammatory cells in the lung tissue was significantly upregulated after LPS administration, and 20 mg/kg of **4c** treatment could reduce the percentage of LCA positive inflammatory cells more significantly than oridonin. In addition, the expression of the genes IL-6 and TNF-α in lung tissue was detected by qPCR. Oridonin and **4c** significantly reduced the LPS-induced expression of the IL-6 and TNF-α genes, and **4c** was much more effective than oridonin (Figures 7D, E). WB analysis of the proteins extracted from the lung tissues revealed that both oridonin and **4c** significantly inhibited the LPS-induced expression of NLRP3, phosphorylated NF-κB and IL-6, and **4c** was more effective than oridonin (Figures 7F–I). In conclusion, compared with oridonin, **4c** showed much more promise for alleviating ALI induced by LPS in mice.

#### 3.2.8 Molecular docking of compound 4c into NLRP3

**FIGURE 8:**
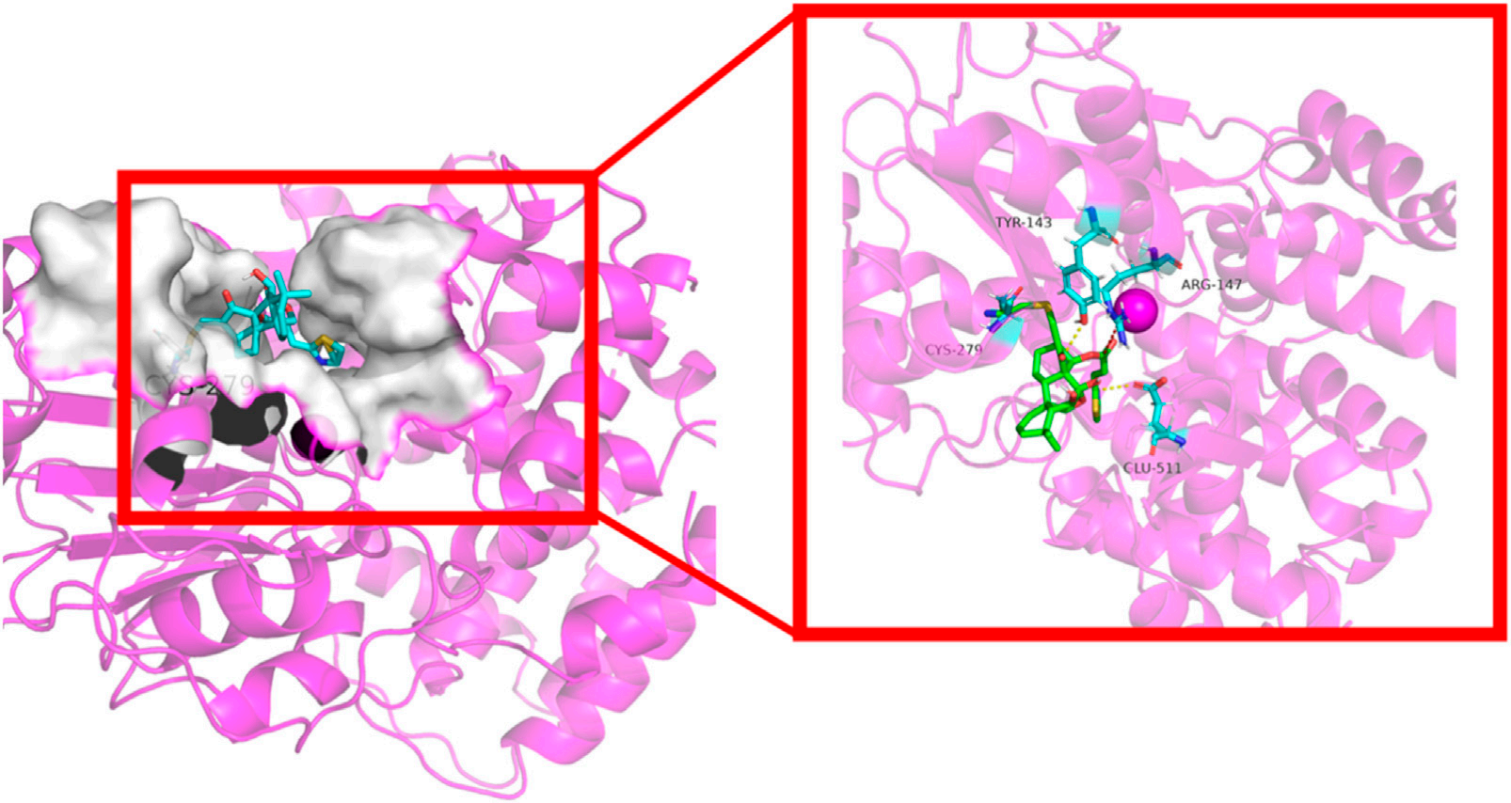
Cartoon representation of compound **4c** bound to the NLRP3 protein (ID: 8etr).

ADFR software was employed to elucidate the binding mode of compound **4c** with the NLRP3 protein, whose crystal structure (ID: 8etr) was downloaded from the Protein Data Bank (https://www.rcsb.org/) (Ravindranath et al., 2015; McBride et al., 2022) The structure of compound **4c** with the lowest energy was prepared with Chem 3D 20.0. The docking results shown in Figure 8 indicate that the *α*,*β*-unsaturated carbonyl unit of compound **4c** could act as a Michael acceptor that targets the thiol group of the residue Cys279 of the NLRP3 protein to form a stable C-S covalent bond, which had been considered during a previous oridonin target investigation (He et al., 2018). Additionally, the protein‒ligand interaction profiler (PLIP) website revealed that compound **4c** could form two hydrogen bonds with residues Tyr143 and Glu511 (marked in yellow), as well as salt bridges with the amino acid residue Arg147 (marked in red). The length of the hydrogen bonds ranged from 3.16 Å to 3.26 Å, implying strong physicochemical forces. These results suggested that the *in silico* predictions were in high accordance with the aforementioned *in vitro* and *in vivo* results and met our initial expectations, which indirectly proved the feasibility of the NLRP3-based structural design strategy.

#### 3.2.9 RNA-seq revealed that 4c regulated inflammatory signaling pathways

**FIGURE 9:**
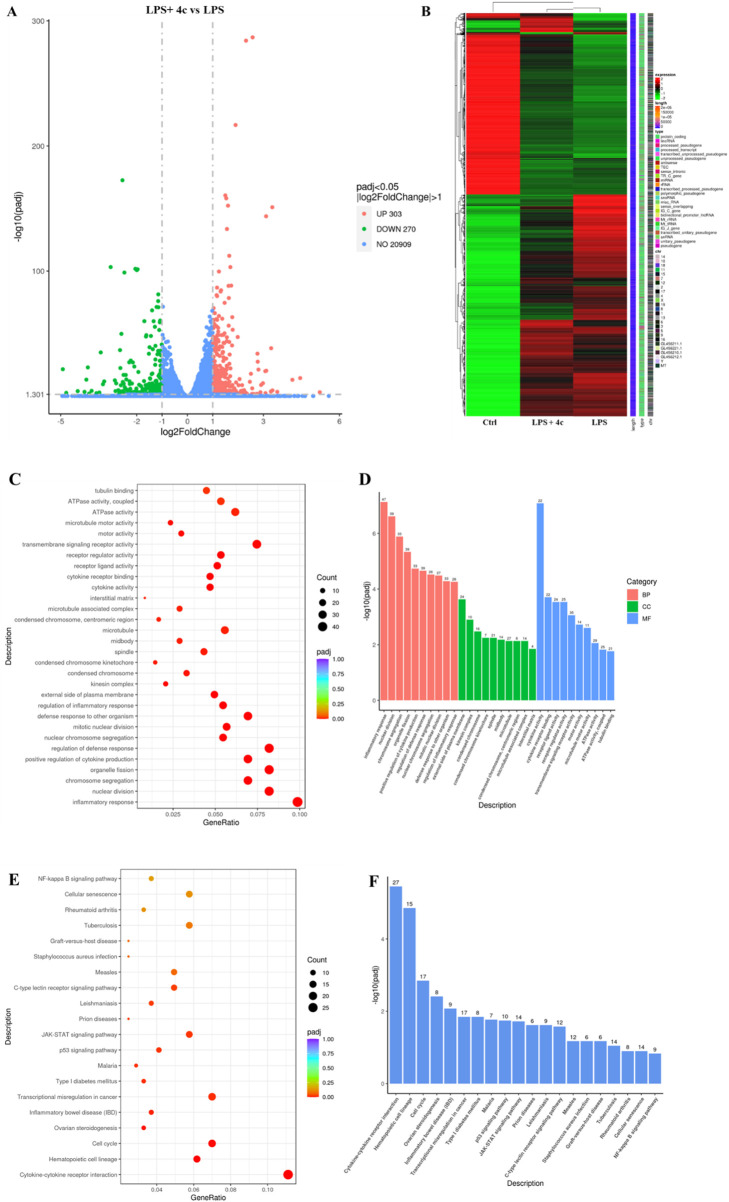
RNA-sequencing revealed that **4c** regulated inflammatory signaling pathways. **(A)**, Volcano plot showing the differentially expressed genes. RAW264.7 cells were pretreated with LPS for 1 h, cocultured with or without 0.312 μM **4c** for 24 h, and then subjected to RNA-seq analysis. **(B)**, Cluster heatmap displaying the differentially expressed genes in RAW264.7 cells in the control group, LPS group, and LPS and **4c** coculture group. **(C, D)**, Gene Ontology (GO) analysis of the differentially expressed genes. **(E, F)**, Kyoto Encyclopedia of Genes and Genomes (KEGG) analysis of the differentially expressed genes.

Both the *in vitro* and *in vivo* assays verified that the anti-inflammatory effects of **4c** occurred via modulation of the NLRP3 inflammasome and the NF-κB pathway. However, the specific mechanism is still not clear. Therefore, RNA-seq analysis was conducted to determine the genes that were differentially expressed in RAW264.7 cells after **4c** treatment. As shown in Figure 9A, the expression of 573 genes was significantly altered after treatment with **4c**, among which 303 were upregulated and 270 were downregulated. Through cluster heatmap analysis, we found that **4c** could significantly reverse LPS-induced gene regulation (Figure 9B). In addition, Gene Ontology (GO) and Kyoto Encyclopedia of Genes and Genomes (KEGG) analyses revealed that inflammation-associated signaling pathways were significantly affected after **4c** treatment (Figures 9C–F).

#### 3.2.10 Validation of the target genes regulated by 4c

**FIGURE 10:**
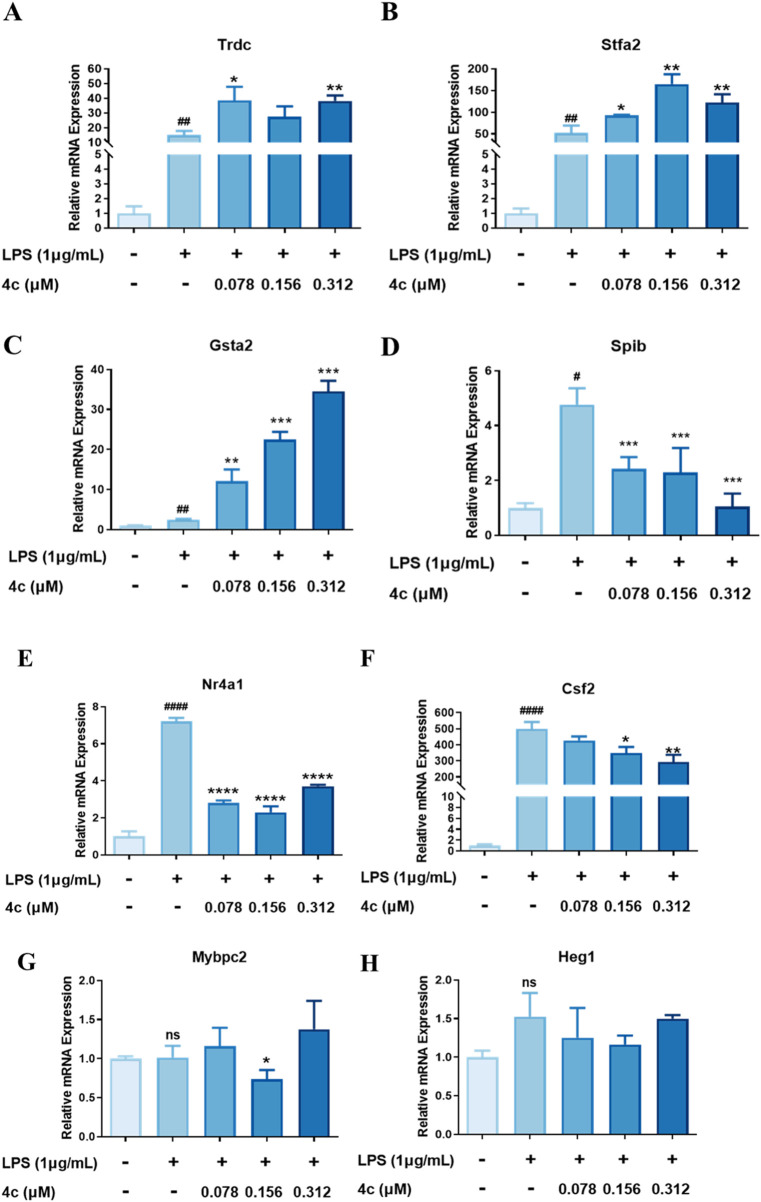
Validation of the target genes regulated by **4c**. **(A‒C)**, qPCR validation of the upregulated genes. **(D‒H)**, qPCR validation of the downregulated genes. RAW264.7 cells were stimulated with 1 μg/mL LPS for 1 h, cocultured with **4c** for 24 h, and then subjected to a qPCR assay. All the results are expressed as the average value ± SD of three independent experiments. # indicates that the difference between the LPS and control groups is significant; and * indicates that the difference between the oridonin derivative and LPS groups is significant. Student’s t-test was used to calculate the significance, ####and ****, p < 0.0001; ### and ***, p < 0.001. ## and **, p < 0.01. # and *, p < 0.05.

On the basis of the aforementioned RNA-seq results, we further used a qPCR technique to verify the top upregulated and downregulated genes (Table 2). The expression of the top 3 upregulated genes, Trdc, Stfa2 and Gsta2, was significantly inhibited after the **4c** treatment (Figures 10A–C). The most downregulated gene, Spib (Figure 10D), and 2 more genes, Nr4a1 and Csf2 (Figures 10E, F), were confirmed to be downregulated after **4c** treatment in a dose-dependent manner. However, the expression of Mybpc2 and Heg1 was not significantly affected by **4c** treatment (Figures 10G, H). For the first time, it was verified that Trdc, Stfa2, Gsta2, Spib, Csf2 and Nr4a1 are significantly regulated by oridonin derivatives.

**TABLE 2:** Genes with significantly upregulated or downregulated expression after treatment with **4c**.

Upregulated genes	Downregulated genes
Trdc	Spib
Stfa2	Mybpc2
Gsta2	Heg1
A530064D06Rik	BE692007
Myh7b	Unc93a
II1f9	Ms4a6c
Gm5483	-
Nqo1	Nr4a1
Iqscc3	-
Trem2	Csf2

## 4 Discussion

### 4.1 SAR study of the oridonin analogs

A novel collection of oridonin hybrids coupled with various acids, including benzoic acid nitrogen mustard, adamantane-1-carboxylic acid and FDA-approved NSAIDs (salicylate, ibuprofen, ketoprofen, and probenecid), were designed and synthesized. The NO production screening assay suggested that removing the OH group at C-1 of oridonin from hit compound 4 could increase the anti-inflammatory activity. Compared with oridonin, further esterification of the C-14 hydroxyl group of **4** also improved the inflammatory activity, especially with compounds **4b** and **4c**. Unfortunately, the further rearrangement of 3 to 6,20-epoxy ent-kaurane diterpenoid scaffold **5** induced by DAST resulted in decreased anti-inflammatory activity, as shown in Table 1, implying that the 7,20-epoxy ent-kaurane diterpenoid scaffold is more favorable. Moreover, oridonin analogs conjugated to NSAIDs showed decreased anti-inflammatory activity, suggesting that there were no synergistic effects after hybridization.

### 4.2 Compound 4c inhibited inflammation by regulating the NF-κB/NLRP3 axis

Inflammasome activation is a tightly regulated inflammatory process, and the activation of the NLRP3 inflammasome consists of priming and activation stages (Swanson et al., 2019). In the priming stage, Toll-like receptors are stimulated by activated cytokines or exogenous pathogens, which activate the NF-κB signaling pathway, leading to increased expression of NLRP3 and pro-IL-1β. In the activation stage, the NLRP3 inflammasome is assembled and mediates the cleavage of pro-caspase-1 into activated caspase-1, which leads to cleavage of pro-IL-18 and pro-IL-1β and results in the secretion of inflammatory cytokines (Jo et al., 2016; Pang et al., 2023; Kelley et al., 2019). Oridonin was reported to covalently bind to Cys279 in the NACHT domain of NLRP3 and block the interaction between NLRP3 and NEK7 to inhibit NLRP3 inflammasome assembly and activation (He et al., 2018). In our study, oridonin derivative **4c** inhibited the NF-κB signaling pathway, the expression of NLRP3 and the secretion of the cytokines TNF-α and IL-6, indicating that **4c** regulated both the priming and activation stages of NLRP3 inflammasome activation. Overall, via regulation of the NF-κB/NLRP3 axis, oridonin derivative **4c** exhibited much greater anti-inflammatory activity than its scaffold oridonin.

### 4.3 RNA-seq analysis suggests the potential anti-inflammatory targets of 4c

To investigate the biological pathways and specific mechanisms by which **4c** regulates inflammation, RNA-seq analysis was conducted. GO analysis indicated that **4c** significantly regulated the biological processes (BP) in RAW264.7 cells and that the most significantly regulated pathway was the inflammatory response. KEGG analysis suggested that the most significantly regulated signaling pathways were the cytokine‒cytokine receptor interaction, hematopoietic cell lineage and cell cycle pathways. Validation of the RNA-seq results revealed that **4c** could upregulate Trdc, Stfa2 and Gsta2 and downregulate Spib, Csf2 and Nr4a1. Moreover, Trdc has been predicted to be involved in the immune response, such as antigen binding and immunoglobulin receptor binding (Malinarich et al., 2010). Stfa2 is an active cysteine protease inhibitor and plays key roles in epidermal development and maintenance (Mezzapesa et al., 2019; Krunic et al., 2013). Gsta2 was found to protect against cell cycle arrest and apoptosis in colon cancer cells (Xie et al., 2005). Spib has been proven to be involved in immature B-cell differentiation and macrophage differentiation (Horiuchi et al., 2023; Lefebvre et al., 2005; Su et al., 1996). Csf2 reportedly modulates inflammation and regulates macrophage polarization (Saita et al., 2022; Li et al., 2022; Gilchrist et al., 2021). Nr4a1 was noted to mediate NK cell dysfunction in hepatocellular carcinoma via the IFN-γ/p-STAT1/IRF1 pathway (Yu et al., 2023). In summary, **4c** was shown to regulate the inflammation-associated genes Trdc, Spib, Csf2 and Nr4a1, which might be potential targets through which **4c** exerts its anti-inflammatory activity; these genes could be investigated in future research. Moreover, the potential anti-inflammatory functions of Stfa2 and Gsta2 could also be investigated and validated in future research.

## 5 Conclusion

In summary, a total of 14 oridonin derivatives were designed and synthesized, and their *in vitro* anti-inflammatory activities were evaluated. The compounds with the 7,20-epoxy ent-kaurane diterpenoid scaffold (series 4) showed more promising efficacy than did the 6,20-epoxy ent-kaurane diterpenoid scaffold (series 5); in particular, compound **4c**, bearing an (E)-3-(thiazol-2-yl) acrylate moiety, showed approximately 17-fold greater anti-inflammatory activity than oridonin did. Additionally, the oridonin hybrids generated by coupling with NSAIDs such as salicylate, ibuprofen, ketoprofen, and probenecid did not show synergistic inhibitory effects on LPS-induced NO production in RAW264.7 cells. The IC_50_ values of the most promising compound, **4c**, against the secretion of IL-1β and IL-6 were 0.21 ± 0.02 μM and 0.21 ± 0.03 μM, respectively. A further qPCR study at the mRNA level indicated that **4c** significantly inhibited the LPS-stimulated expression of inflammatory genes, including IL-6, COX-2 and IL-1β, in a dose-dependent manner, but not iNOS and TNF-α. Furthermore, compound **4c** dose-dependently inhibited the LPS-induced expression of inflammatory proteins, including phosphorylated NF-κB, phosphorylated IκB, NLRP3, IL-6 and iNOS. More importantly, **4c** alleviated the symptoms of ALI in mice. The RT‒qPCR results showed that **4c** reduced the LPS-induced expression of IL-6 and TNF-α in lung tissue at the mRNA level; moreover, the WB results indicated that **4c** could significantly inhibit the production of NLRP3, phosphorylated NF-κB and IL-6 in lung tissues. Docking simulations were conducted to position compound **4c** into the NLRP3 binding site to predict the binding mode, and it was suggested that the *α*,*β*-unsaturated carbonyl moiety could act as a Michael acceptor that targets the thiol group of the residue Cys279 of the NLRP3 protein to form a stable C-S covalent bond. The results of RNA-seq and RT‒qPCR revealed that **4c** could upregulate the genes Trdc, Stfa2 and Gsta2 and downregulate the genes Spib, Csf2 and Nr4a1. In conclusion, this series of oridonin analogs could be used as promising lead candidates for the treatment of NLRP3-driven disorders.

## Data Availability

The datasets presented in this study can be found in online repositories. The names of the repository/repositories and accession number(s) can be found in the article/Supplementary Material.
