# Supplementary material for: In vitro and in vivo characterization of oridonin analogs as anti-inflammatory agents that regulate the NF-κB and NLRP3 inflammasome axis
Source: Front Pharmacol. 2025 Feb 27;16:1512740. doi: 10.3389/fphar.2025.1512740 (PMC11903421; doi:10.3389/fphar.2025.1512740)
Supplement: Supplementary file 1 [file DataSheet1.docx]

Supplementary Material

# Supplementary Data

## General procedure for the preparation of oridonin hybrids 4a-4e and 4g-4j

A mixture of compound **4** (480 mg, 1.40 mmol), EDCI (200 mg, 1.04 mmol), HOBt (254 mg, 1.04 mmol), DMAP (catalytic amount), and the corresponding acid (112 mg, 0.62 mmol) in DCM was stirred at room temperature overnight. Afterward, the solution was washed with 10% HCl. The organic layer was subsequently dried over anhydrous MgSO4, filtered, and evaporated in vacuo to obtain a crude mixture. The crude mixture was purified via flash column chromatography on silica gel with petroleum ether/ethyl acetate (4:1) as the eluent to afford the targeted oridonin hybrids **4a**-**4e** and **4g**-**4j**.

***(6aR,9S,11bR,14R)-5,6-Dihydroxy-4,4-dimethyl-8-methylene-7-oxo-4,4a,5,6,7,8,9,10,11,11a-decahydro-3H-6,11b-(epoxymethano)-6a,9-methanocyclohepta[a]naphthalen-14-yl acrylate (4b)***

Yield: 37%. 1H NMR (400 MHz, CDCl3): δ 6.38 (dd, J = 1.22, 17.36 Hz, 1H), 6.20-6.22 (m, 1H), 5.98-6.10 (m, 1H), 5.92-5.94 (m, 1H), 5.85 (dd, J = 1.22, 10.52 Hz, 1H), 5.81 (ddd, J = 2.08, 6.24, 10.27 Hz, 1H), 5.54-5.58 (m, 2H), 5.21 (dd, J = 2.57, 10.39 Hz, 1H), 4.08-4.10 (m, 1H), 3.96-4.01 (m, 1H), 3.87-3.93 (m, 1H), 3.80 (dd, J = 8.31, 11.49 Hz, 1H), 3.18 (d, J = 10.03 Hz, 1H), 2.56-2.68 (m, 1H), 1.91-1.99 (m, 3H), 1.80-1.89 (m, 1H), 1.61-1.71 (m, 3H), 1.52 (dd, J = 1.22, 8.31 Hz, 1H), 1.26 (s, 1H), 1.15-1.18 (m, 3H), 1.04 (s, 3H);13C NMR (100 MHz, CDCl3): δ 164.7, 149.6, 132.2, 130.9, 127.9, 124.2, 121.4, 96.5, 75.7, 73.5, 65.5, 61.8, 58.2, 53.3, 41.4, 41.0, 38.5, 32.4, 30.8, 30.3, 21.7, 17.8; HRMS m/z calcd for [C23H28O6+H]+ 401.1964, found 401.2029.

***5,6-Dihydroxy-4,4-dimethyl-8-methylene-7-oxo-4,4a,5,6,7,8,9,10,11,11a-decahydro-3H-6,11b-(epoxymethano)-6a,9-methanocyclohepta[a]naphthalen-14-yl (E)-3-(thiazol-2-yl)acrylate (4c)***

Yield: 43%. 1H NMR (600 MHz, CDCl3): δ ppm 1.03 (s, 3 H) 1.16 (s, 3 H) 1.52 (d, J = 8.36 Hz, 1 H) 1.62-1.72 (m, 2 H) 1.80-1.90 (m, 2 H) 1.92-1.96 (m, 2 H) 2.59-2.67 (m, 1 H) 3.18 (d, J = 9.26 Hz, 1 H) 3.78 (dd, J = 11.63, 8.54 Hz, 1 H) 3.90 (d, J =10.90 Hz, 1 H) 3.99 (d, J = 10.17 Hz, 1 H) 4.10 (s, 1 H) 5.22 (dd, J = 10.26, 2.63 Hz, 1 H) 5.58 (t, J = 5.90 Hz, 2 H) 5.80 (ddd, J = 10.13, 6.31, 1.54 Hz, 1 H) 6.04 (s, 1 H) 6.23 (s, 1 H) 6.63 (d, J = 15.80 Hz, 1 H) 7.43 (d, J = 3.09 Hz, 1 H) 7.74 (d, J = 15.99 Hz, 1 H) 7.90 (d, J = 3.09 Hz, 1 H); 13C NMR (150 MHz, CDCl3): δ 163.2, 144.8, 136.9, 130.9, 124.2, 122.4, 121.7, 121.5, 96.5, 75.4, 73.7, 65.5, 62.0, 57.9, 53.3, 41.6, 41.0, 38.6, 32.4, 30.8, 30.3, 21.7, 17.9; HRMS m/z calcd for [C26H29NO6S+H]+ 484.1716, found 484.1761.

***5,6-Dihydroxy-4,4-dimethyl-8-methylene-7-oxo-4,4a,5,6,7,8,9,10,11,11a-decahydro-3H-6,11b-(epoxymethano)-6a,9-methanocyclohepta[a]naphthalen-14-yl (2E,4E)-hexa-2,4-dienoate (4d)***

Yield: 78%. 1H NMR (600 MHz, CDCl3): δ 7.17 (dd, J = 10.17, 15.26 Hz, 1H), 6.05-6.20 (m, 3H), 5.85 (s, 1H), 5.74-5.82 (m, 1H), 5.67 (d, J = 15.26 Hz, 1H), 5.50-5.59 (m, 2H), 5.19 (dd, J = 2.72, 10.17 Hz, 1H), 4.31 (s, 1H), 3.95 (d, J = 10.17 Hz, 1H), 3.85-3.90 (m, 1H), 3.76-3.82 (m, 1H), 3.17 (d, J = 9.45 Hz, 1H), 2.58-2.64 (m, 1H), 1.87-1.99 (m, 4H), 1.82 (d, J = 5.99 Hz, 3H), 1.59-1.67 (m, 2H), 1.51 (d, J = 8.17 Hz, 1H), 1.15 (s, 3H), 1.02 (s, 3H); 13C NMR (150 MHz, CDCl3); δ 205.1, 165.5, 149.6, 146.7, 141.2, 130.9, 129.5, 124.1, 121.3, 117.7, 96.6, 76.0, 73.3, 65.5, 61.7, 58.5, 53.3, 41.3, 41.1, 38.5, 32.4, 30.7, 30.2, 21.7, 18.7, 17.8; HRMS m/z calcd for [C26H32O6+H]+ 441.2277, found 441.2275.

***5,6-Dihydroxy-4,4-dimethyl-8-methylene-7-oxo-4,4a,5,6,7,8,9,10,11,11a-decahydro-3H-6,11b-(epoxymethano)-6a,9-methanocyclohepta[a]naphthalen-14-yl 2-hydroxybenzoate (4g)***

Yield: 44%. 1H NMR (400 MHz, CDCl3): δ 10.48 (s, 1H), 7.70 (dd, J = 1.42, 7.98 Hz, 1H), 7.42-7.49 (m, 1H), 6.97 (d, J = 8.41 Hz, 1H), 6.86 (t, J = 7.42 Hz, 1H), 6.30 (s, 1H), 6.21 (s, 1H), 5.86 (ddd, J = 1.61, 6.22, 9.99 Hz, 1H), 5.64 (s, 1H), 5.27 (dd, J = 2.54, 10.21 Hz, 1H), 4.05 (d, J = 10.27 Hz, 1H), 3.95 (d, J = 10.14 Hz, 1H), 3.80 (br. s., 1H), 3.28 (d, J = 9.28 Hz, 1H), 2.65-2.74 (m, 1H), 2.00 (d, J = 3.46 Hz, 1H), 1.97 (br. s., 1H), 1.91 (d, J = 6.19 Hz, 1H), 1.74 (dd, J = 3.96, 6.80 Hz, 1H), 1.57 (d, J = 8.66 Hz, 1H), 1.29 (br. s., 4H), 1.20 (s, 3H), 1.07 (s, 3H); 13C NMR (101 MHz, CDCl3): δ ppm 18.00, 21.67, 30.34, 30.79, 32.38, 38.65, 40.99, 41.71, 53.41, 57.71, 62.13, 73.92, 75.26, 96.38, 117.56, 119.38, 124.24, 130.37, 131.03, 135.85, 149.64, 161.37; HRMS m/z calcd for [C27H30O7+H]+ 467.2070, found 467.2092.

***5,6-Dihydroxy-4,4-dimethyl-8-methylene-7-oxo-4,4a,5,6,7,8,9,10,11,11a-decahydro-3H-6,11b-(epoxymethano)-6a,9-methanocyclohepta[a]naphthalen-14-yl (2R)-2-(4-isobutylphenyl)propanoate (4h)***

Yield: 27%. 1H NMR (400 MHz, CDCl3): δ ppm 0.88-0.95 (m, 13 H) 1.06 (s, 2 H) 1.18 (s, 2 H) 1.45-1.51 (m, 2 H) 1.54 (d, J = 7.18 Hz, 4 H) 1.82-1.96 (m, 3 H) 2.05 (d, J = 5.20 Hz, 1 H) 2.43-2.50 (m, 3 H) 2.92 (d, J = 9.40 Hz, 1 H) 3.71-3.78 (m, 1 H) 3.90 (d, J = 10.39 Hz, 1 H) 4.00 (d, J = 10.27 Hz, 1 H) 5.18-5.26 (m, 1 H) 5.80-5.85 (m, 1 H) 5.99 (s, 1 H) 7.04-7.09 (m, 1 H) 7.10-7.16 (m, 3 H) 7.26 (d, J=8.04 Hz, 2 H); 13C NMR (101 MHz, CDCl3): δ 173.2, 149.3, 140.8, 130.9, 129.4, 127.1, 124.1, 120.8, 96.6, 76.0, 73.3, 61.7, 58.4, 53.2, 45.2, 45.0, 41.1, 40.9, 38.4, 32.4, 30.7, 30.2, 29.9, 22.4, 21.7, 17.8, 17.6, -3.7; HRMS m/z calcd for [C33H42O6+H]+ 533.2981, found 533.2923.

***5,6-Dihydroxy-4,4-dimethyl-8-methylene-7-oxo-4,4a,5,6,7,8,9,10,11,11a-decahydro-3H-6,11b-(epoxymethano)-6a,9-methanocyclohepta[a]naphthalen-14-yl 4-(N,N-dipropylsulfamoyl)benzoate (4i)***

Yield: 21%. 1H NMR (400 MHz, CDCl3): δ 8.03-8.09 (m, J = 8.54 Hz, 2H), 7.79-7.84 (m, J = 8.54 Hz, 2H), 6.27 (s, 1H), 6.18 (s, 1H), 5.83 (ddd, J = 1.73, 6.15, 10.05 Hz, 1H), 5.58 (d, J = 11.88 Hz, 1H), 5.24 (dd, J = 2.29, 10.21 Hz, 1H), 4.00 (d, J = 10.27 Hz, 1H), 3.93 (d, J = 10.14 Hz, 1H), 3.87 (s, 1H), 3.75 (dd, J = 8.66, 11.88 Hz, 1H), 3.24 (d, J = 9.28 Hz, 1H), 3.03-3.09 (m, 4H), 2.62-2.72 (m, 1H), 1.91-2.03 (m, 3H), 1.81 - 1.91 (m, 1H), 1.67 - 1.76 (m, 2H), 1.64 (s, 2H), 1.51 - 1.57 (m, 4H), 1.16 (s, 3H), 1.03 (s, 3H), 0.86 (t, J = 7.42 Hz, 6H); 13C NMR (101 MHz, CDCl3): δ 205.1, 164.3, 149.8, 144.2, 133.4, 131.0, 130.5, 127.0, 124.2, 121.6, 96.4, 75.4, 74.0, 65.6, 62.2, 57.8, 53.4, 50.0, 41.7, 41.0, 38.6, 32.4, 30.8, 30.4, 22.0, 21.7, 18.0, 11.2; HRMS m/z calcd for [C33H43NO8S+H]+ 614.2709, found 614.2789.

***5,6-Dihydroxy-4,4-dimethyl-8-methylene-7-oxo-4,4a,5,6,7,8,9,10,11,11a-decahydro-3H-6,11b-(epoxymethano)-6a,9-methanocyclohepta[a]naphthalen-14-yl 2-(3-benzoylphenyl)propanoate (4j)***

Yield: 20%. 1H NMR (400 MHz, CDCl3): δ 7.83-7.86 (m, 2H), 7.66-7.70 (m, 2H), 7.62 (d, J = 7.42 Hz, 1H), 7.54 (d, J = 7.79 Hz, 2H), 7.44-7.49 (m, 2H), 6.17 (s, 1H), 5.84 (s, 1H), 5.51 (d, J = 2.47 Hz, 1H), 5.47 (s, 1H), 5.38 (s, 1H), 5.22 (d, J = 2.47 Hz, 1H), 3.94 (d, J = 10.27 Hz, 1H), 3.85 (d, J = 10.27 Hz, 1H), 3.73-3.78 (m, 1H), 3.58 (s, 1H), 3.11 (d, J = 10.02 Hz, 1H), 2.24 (d, J = 7.79 Hz, 1H), 2.05 (d, J = 5.44 Hz, 1H), 1.93 (d, J = 4.70 Hz, 2H), 1.87 (d, J = 6.06 Hz, 1H), 1.67 (s, 1H), 1.64 (d, J = 3.34 Hz, 2H), 1.53 (d, J = 7.18 Hz, 3H), 1.46 (s, 1H), 1.17 (s, 3H), 1.02 (s, 3H); 13C NMR (101 MHz, CDCl3): δ 132.5, 131.5, 130.9, 130.2, 129.3, 129.1, 128.8, 128.3, 124.1, 96.4, 77.2, 76.2, 73.3, 65.4, 58.1, 53.3, 45.8, 41.4, 41.0, 32.3, 29.7, 29.4, 21.7, 17.9; HRMS m/z calcd for [C36H38O7+H]+ 583.2618, found 583.2685.

## General procedure for the preparation of oridonin analogs 5a-5d

A mixture of compounds **3** or **4a**-**4d** (0.152 mmol) and DAST (60 mg, 0.372 mmol) in DCM was stirred at -78 °C for 20 min under a nitrogen atmosphere and then at room temperature for 2 hours. Afterward, the mixture was filtered and evaporated in vacuo to obtain the crude product, which was purified via flash column chromatography on silica gel with petroleum ether/ethyl acetate (4:1) as the eluent to afford the desired oridonin analogs **5** and **5a**-**5d**.

***(5R,6aR,9S,11bR,14R)-4,4-Dimethyl-8-methylene-6,7-dioxo-4,4a,5,6,7,8,9,10,11,11a-decahydro-3H-5,11b-(epoxymethano)-6a,9-methanocyclohepta[a]naphthalen-14-yl acrylate (5b)***

Yield: 68%. 1H NMR (400 MHz, CDCl3): δ 6.38 (dd, J = 1.34, 17.24 Hz, 1H), 6.13 (s, 1H), 5.98-6.07 (m, 1H), 5.77-5.85 (m, 2H), 5.63-5.73 (m, 2H), 5.45 (s, 1H), 4.26-4.33 (m, 2H), 3.83 (d, J = 9.05 Hz, 1H), 3.25 (d, J = 7.34 Hz, 1H), 2.47-2.57 (m, 1H), 2.13-2.19 (m, 1H), 1.84-2.05 (m, 3H), 1.71-1.83 (m, 3H), 1.04 (d, J = 4.16 Hz, 6H); 13C NMR (100 MHz, CDCl3): δ 202.8, 164.9, 147.4, 132.1, 128.8, 127.8, 123.6, 118.9, 82.7, 76.0, 72.1, 65.3, 56.6, 51.5, 42.2, 39.6, 30.4, 30.1, 22.9, 17.7; HRMS m/z calcd for [C23H26O5+Na]+ 405.1678, found 405.1671.

***4,4-Dimethyl-8-methylene-6,7-dioxo-4,4a,5,6,7,8,9,10,11,11a-decahydro-3H-5,11b-(epoxymethano)-6a,9-methanocyclohepta[a]naphthalen-14-yl (E)-3-(thiazol-2-yl)acrylate (5c)***

Yield: 39%. 1H NMR (600 MHz, CDCl3): δ ppm 0.97 (d, J = 5.45 Hz, 7 H) 1.67-1.71 (m, 2 H) 1.75 (s, 1 H) 1.82 (dd, J = 17.62, 6.18 Hz, 1 H) 1.89-1.96 (m, 2 H) 2.03-2.18 (m, 1 H) 2.47 (d, J = 8.72 Hz, 1 H) 3.21 (d, J = 7.45 Hz, 1 H) 3.77 (d, J = 9.08 Hz, 1 H) 4.18-4.31 (m, 2 H) 5.41 (s, 1 H) 5.60 (dd, J = 10.35, 2.54 Hz, 1 H) 5.67-5.79 (m, 2 H) 6.08 (s, 1 H) 6.55 (d, J=15.80 Hz, 1 H) 7.35 (d, J = 3.09 Hz, 1 H) 7.68 (d, J = 15.99 Hz, 1 H) 7.83 (d, J = 3.27 Hz, 1 H); 13C NMR (150 MHz, CDCl3): δ 164.7, 147.4, 144.7, 137.0, 128.8, 123.5, 122.1, 121.6, 119.0, 82.7, 76.4, 72.1, 65.2, 56.5, 51.5, 50.4, 42.3, 39.6, 30.4, 30.1, 22.9, 17.7; HRMS m/z calcd for [C26H27NO5S+H]+ 466.1710, found 466.1709.

***4,4-Dimethyl-8-methylene-6,7-dioxo-4,4a,5,6,7,8,9,10,11,11a-decahydro-3H-5,11b-(epoxymethano)-6a,9-methanocyclohepta[a]naphthalen-14-yl (2E,4E)-hexa-2,4-dienoate (5d)***

Yield: 64%. 1H NMR (600 MHz, CDCl3): δ 7.20 (dd, J = 9.54, 15.35 Hz, 1H), 6.09-6.17 (m, 3H), 5.80 (dd, J = 5.90, 10.08 Hz, 1H), 5.63-5.72 (m, 3H), 5.44 (s, 1H), 4.25-4.35 (m, 2H), 3.82 (d, J = 9.26 Hz, 1H), 3.24 (d, J = 7.45 Hz, 1H), 2.45-2.60 (m, 1H), 2.14 (t, J = 8.17 Hz, 1H), 1.85-2.04 (m, 4H), 1.78-1.84 (m, 4H), 1.70-1.77 (m, 2H), 1.04 (d, J = 5.81 Hz, 6H); 13C NMR (150 MHz, CDCl3): δ 203.0, 199.6, 165.9, 147.6, 146.3, 140.1, 129.7, 128.7, 123.6, 118.7, 118.2, 82.7, 75.7, 72.1, 65.3, 56.7, 51.5, 50.4, 42.3, 39.6, 30.4, 30.1, 22.9, 18.7, 17.7; HRMS m/z calcd for [C26H30O5+H]+ 423.2093, found 423.2182.

# Supplementary Figures and Tables

## Supplementary Figures

**Supplementary Figure 1.** 1H NMR spectra of

***(3S,3aR,3a1R,6aS,7S,11S,11aS)-7,11-dihydroxy-5,5,8,8-tetramethyl-15-methylenedecahydro-2H-6a,11a-(epoxymethano)-3,3a1-ethanophenanthro[1,10-de] [1,3] dioxin-14-one* (1)**

***
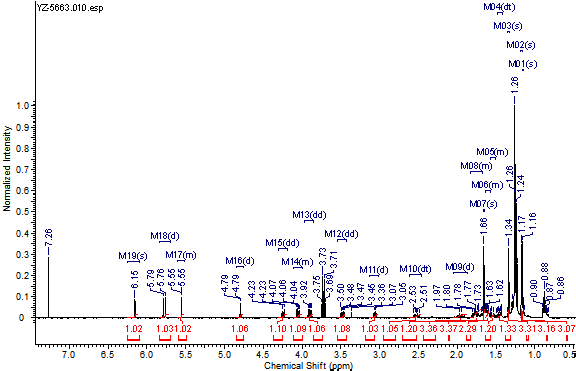
***

****Supplementary Figure 2.** ^1^H NMR spectra of

***(3S,3aR,3a1R,6aS,7S,11S,11aS)-7-hydroxy-5,5,8,8-tetramethyl-15-methylene-14-oxodecahydro-2H-6a,11a-(epoxymethano)-3,3a1-ethanophenanthro[1,10-de] [1,3] dioxin-11-yl methane sulfonate (2)***


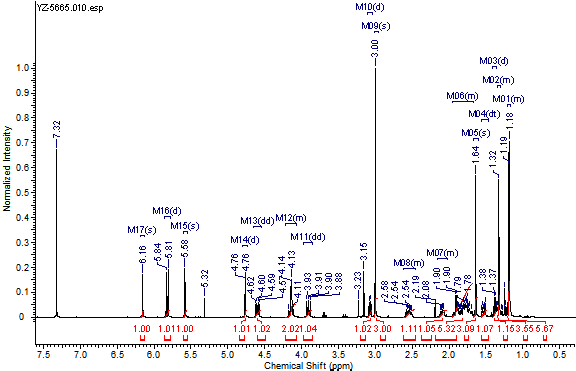


**Supplementary Figure 3.** ^1^H NMR spectra of

***(3S,3aR,3a1R,6aS,7S,11aR)-7-hydroxy-5,5,8,8-tetramethyl-15-methylene-1,3,3a,7,7a,8,9,11b-octahydro-2H-6a,11a-(epoxymethano)-3,3a1-ethanophenanthro[1,10-de] [1,3] dioxin-14-one (3)***

**
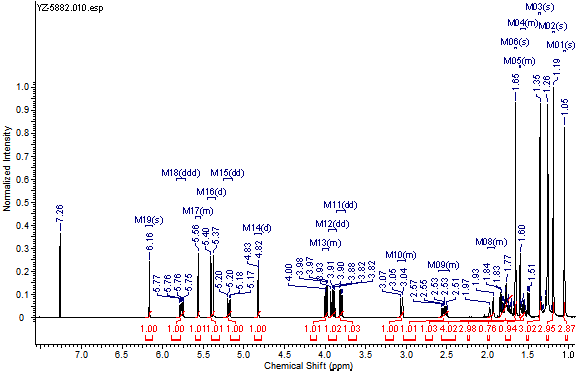
**

**Supplementary Figure 4.** ^1^H and ^13^C NMR spectra of

***5,6,14-trihydroxy-4,4-dimethyl-8-methylene-4,4a,5,6,9,10,11,11a-octahydro-3H-6,11b-(epoxymethano)-6a,9-methanocyclohepta[a]naphthalen-7(8H)-one (4)***

**Supplementary Figure 5.** ^1^H and ^13^C NMR spectra of

***5,6-dihydroxy-4,4-dimethyl-8-methylene-7-oxo-4,4a,5,6,7,8,9,10,11,11a-decahydro-3H-6,11b-(epoxymethano)-6a,9-methanocyclohepta[a]naphthalen-14-yl adamantane-1-carboxylate (4a)***

**Supplementary Figure 6.** ^1^H and ^13^C NMR spectra of

***(6aR,9S,11bR,14R)-5,6-dihydroxy-4,4-dimethyl-8-methylene-7-oxo-4,4a,5,6,7,8,9,10,11,11a-decahydro-3H-6,11b-(epoxymethano)-6a,9-methanocyclohepta[a]naphthalen-14-yl acrylate (4b).***

**Supplementary Figure 7.** ^1^H and ^13^C NMR spectra of

***5,6-dihydroxy-4,4-dimethyl-8-methylene-7-oxo-4,4a,5,6,7,8,9,10,11,11a-decahydro-3H-6,11b-(epoxymethano)-6a,9-methanocyclohepta[a]naphthalen-14-yl (E)-3-(thiazol-2-yl)acrylate (4c).***

**Supplementary Figure 8.** 1H and 13C NMR spectra of

***5,6-dihydroxy-4,4-dimethyl-8-methylene-7-oxo-4,4a,5,6,7,8,9,10,11,11a-decahydro-3H-6,11b-(epoxymethano)-6a,9-methanocyclohepta[a]naphthalen-14-yl (2E,4E)-hexa-2,4-dienoate (4d)***

**Supplementary Figure 9.** ^1^H and ^13^C NMR spectra of

***5,6-dihydroxy-4,4-dimethyl-8-methylene-7-oxo-4,4a,5,6,7,8,9,10,11,11a-decahydro-3H-6,11b-(epoxymethano)-6a,9-methanocyclohepta[a]naphthalen-14-yl 2-(3,4-dimethoxyphenyl) acetate (4e)***

**Supplementary Figure 10.** ^1^H and ^13^C NMR spectra of

***5,6-dihydroxy-4,4-dimethyl-8-methylene-7-oxo-4,4a,5,6,7,8,9,10,11,11a-decahydro-3H-6,11b-(epoxymethano)-6a,9-methanocyclohepta[a]naphthalen-14-yl 2-hydroxybenzoate (4g)***

**Supplementary Figure 11.** ^1^H and ^13^C NMR spectra of

***5,6-dihydroxy-4,4-dimethyl-8-methylene-7-oxo-4,4a,5,6,7,8,9,10,11,11a-decahydro-3H-6,11b-(epoxymethano)-6a,9-methanocyclohepta[a]naphthalen-14-yl (2R)-2-(4-isobutylphenyl)propanoate (4h)***

**Supplementary Figure 12.** ^1^H and ^13^C NMR spectra of

***5,6-dihydroxy-4,4-dimethyl-8-methylene-7-oxo-4,4a,5,6,7,8,9,10,11,11a-decahydro-3H-6,11b-(epoxymethano)-6a,9-methanocyclohepta[a]naphthalen-14-yl 4-(N,N-dipropylsulfamoyl)benzoate (4i)***

**Supplementary Figure 13.** ^1^H and ^13^C NMR spectra of ***5,6-dihydroxy-4,4-dimethyl-8-methylene-7-oxo-4,4a,5,6,7,8,9,10,11,11a-decahydro-3H-6,11b-(epoxymethano)-6a,9-methanocyclohepta[a]naphthalen-14-yl 2-(3-benzoylphenyl)propanoate (4j)***

**Supplementary Figure 14.** ^1^H and ^13^C NMR spectra of

***14-hydroxy-4,4-dimethyl-8-methylene-3,4,4a,5,9,10,11,11a-octahydro-6H-5,11b-(epoxymethano)-6a,9-methanocyclohepta[a]naphthalene-6,7(8H)-dione (5).***

**Supplementary Figure 15.** ^1^H and ^13^C NMR spectra of

***4,4-dimethyl-8-methylene-6,7-dioxo-4,4a,5,6,7,8,9,10,11,11a-decahydro-3H-5,11b-(epoxymethano)-6a,9-methanocyclohepta[a]naphthalen-14-yl adamantane-1-carboxylate (5a).***

**Supplementary Figure 16.** ^1^H and ^13^C NMR spectra of

***(5R,6aR,9S,11bR,14R)-4,4-dimethyl-8-methylene-6,7-dioxo-4,4a,5,6,7,8,9,10,11,11a-decahydro-3H-5,11b-(epoxymethano)-6a,9-methanocyclohepta[a]naphthalen-14-yl acrylate (5b).***

**Supplementary Figure 17.** ^1^H and ^13^C NMR spectra of

***4,4-dimethyl-8-methylene-6,7-dioxo-4,4a,5,6,7,8,9,10,11,11a-decahydro-3H-5,11b-(epoxymethano)-6a,9-methanocyclohepta[a]naphthalen-14-yl (E)-3-(thiazol-2-yl)acrylate (5c).***

**Supplementary Figure 18.** ^1^H and ^13^C NMR spectra of

***4,4-dimethyl-8-methylene-6,7-dioxo-4,4a,5,6,7,8,9,10,11,11a-decahydro-3H-5,11b-(epoxymethano)-6a,9-methanocyclohepta[a]naphthalen-14-yl (2E,4E)-hexa-2,4-dienoate (5d)***

**Supplementary Figure 19.** ^1^H and ^13^C NMR spectra of

***4,4-dimethyl-8-methylene-6,7-dioxo-4,4a,5,6,7,8,9,10,11,11a-decahydro-3H-5,11b-(epoxymethano)-6a,9-methanocyclohepta[a]naphthalen-14-yl 4-(bis(2-chloroethyl) amino) benzoate (5f)***

**Supplementary Figure 20.** HRMS spectra of

***5,6,14-trihydroxy-4,4-dimethyl-8-methylene-4,4a,5,6,9,10,11,11a-octahydro-3H-6,11b-(epoxymethano)-6a,9-methanocyclohepta[a]naphthalen-7(8H)-one (4)***

**Supplementary Figure 21.** HRMS spectra of

***5,6-dihydroxy-4,4-dimethyl-8-methylene-7-oxo-4,4a,5,6,7,8,9,10,11,11a-decahydro-3H-6,11b-(epoxymethano)-6a,9-methanocyclohepta[a]naphthalen-14-yl adamantane-1-carboxylate (4a)***

**Supplementary Figure 22.** HRMS spectra of

***(6aR,9S,11bR,14R)-5,6-dihydroxy-4,4-dimethyl-8-methylene-7-oxo-4,4a,5,6,7,8,9,10,11,11a-decahydro-3H-6,11b-(epoxymethano)-6a,9-methanocyclohepta[a]naphthalen-14-yl acrylate (4b).***

**Supplementary Figure 23.** HRMS spectra of

***5,6-dihydroxy-4,4-dimethyl-8-methylene-7-oxo-4,4a,5,6,7,8,9,10,11,11a-decahydro-3H-6,11b-(epoxymethano)-6a,9-methanocyclohepta[a]naphthalen-14-yl (E)-3-(thiazol-2-yl) acrylate (4c).***

**Supplementary Figure 24.** HRMS spectra of

***5,6-dihydroxy-4,4-dimethyl-8-methylene-7-oxo-4,4a,5,6,7,8,9,10,11,11a-decahydro-3H-6,11b-(epoxymethano)-6a,9-methanocyclohepta[a]naphthalen-14-yl (2E,4E)-hexa-2,4-dienoate (4d)***

**Supplementary Figure 25.** HRMS spectra of

***5,6-dihydroxy-4,4-dimethyl-8-methylene-7-oxo-4,4a,5,6,7,8,9,10,11,11a-decahydro-3H-6,11b-(epoxymethano)-6a,9-methanocyclohepta[a]naphthalen-14-yl 2-(3,4-dimethoxyphenyl) acetate (4e)***

**Supplementary Figure 26.** HRMS spectra of

***5,6-dihydroxy-4,4-dimethyl-8-methylene-7-oxo-4,4a,5,6,7,8,9,10,11,11a-decahydro-3H-6,11b-(epoxymethano)-6a,9-methanocyclohepta[a]naphthalen-14-yl 2-hydroxybenzoate (4g)***

**Supplementary Figure 27.** HRMS spectra of

***5,6-dihydroxy-4,4-dimethyl-8-methylene-7-oxo-4,4a,5,6,7,8,9,10,11,11a-decahydro-3H-6,11b-(epoxymethano)-6a,9-methanocyclohepta[a]naphthalen-14-yl (2R)-2-(4-isobutylphenyl)propanoate (4h)***

**Supplementary Figure 28.** HRMS spectra of

***5,6-dihydroxy-4,4-dimethyl-8-methylene-7-oxo-4,4a,5,6,7,8,9,10,11,11a-decahydro-3H-6,11b-(epoxymethano)-6a,9-methanocyclohepta[a]naphthalen-14-yl 4-(N,N-dipropylsulfamoyl)benzoate (4i)***

**Figure S29.** HRMS spectra of

***5,6-dihydroxy-4,4-dimethyl-8-methylene-7-oxo-4,4a,5,6,7,8,9,10,11,11a-decahydro-3H-6,11b-(epoxymethano)-6a,9-methanocyclohepta[a]naphthalen-14-yl 2-(3-benzoylphenyl)propanoate (4j)***

**Supplementary Figure 30.** HRMS spectra of

***14-hydroxy-4,4-dimethyl-8-methylene-3,4,4a,5,9,10,11,11a-octahydro-6H-5,11b-(epoxymethano)-6a,9-methanocyclohepta[a]naphthalene-6,7(8H)-dione (5)***

**Figure S31.** HRMS spectra of

***4,4-dimethyl-8-methylene-6,7-dioxo-4,4a,5,6,7,8,9,10,11,11a-decahydro-3H-5,11b-(epoxymethano)-6a,9-methanocyclohepta[a]naphthalen-14-yl adamantane-1-carboxylate (5a).***

**Supplementary Figure 32.** HRMS spectra of

***(5R,6aR,9S,11bR,14R)-4,4-dimethyl-8-methylene-6,7-dioxo-4,4a,5,6,7,8,9,10,11,11a-decahydro-3H-5,11b-(epoxymethano)-6a,9-methanocyclohepta[a]naphthalen-14-yl acrylate (5b).***

**Supplementary Figure 33.** HNMR spectra of

***4,4-dimethyl-8-methylene-6,7-dioxo-4,4a,5,6,7,8,9,10,11,11a-decahydro-3H-5,11b-(epoxymethano)-6a,9-methanocyclohepta[a]naphthalen-14-yl (E)-3-(thiazol-2-yl)acrylate (5c).***

**Supplementary Figure 34.** HNMR spectra of

***4,4-dimethyl-8-methylene-6,7-dioxo-4,4a,5,6,7,8,9,10,11,11a-decahydro-3H-5,11b-(epoxymethano)-6a,9-methanocyclohepta[a]naphthalen-14-yl (2E,4E)-hexa-2,4-dienoate (5d)***

**Figure S35.** HNMR spectra of

***4,4-dimethyl-8-methylene-6,7-dioxo-4,4a,5,6,7,8,9,10,11,11a-decahydro-3H-5,11b-(epoxymethano)-6a,9-methanocyclohepta[a]naphthalen-14-yl 4-(bis(2-chloroethyl) amino) benzoate (5f)***

## Supplementary Tables

**Supplementary Table 1** Primer sequences used in qPCR assay

| Gene | Primer sequence(5’ to 3’ ) |
| --- | --- |
| IL-6 | F: CACTCACGGCAAATTCAACGGCA |
|  | R: TAACGCACTAGGTTTGCCGA |
| IL-1β | F: CCTGGGCTGTCCTGATGAGAG |
|  | R: TCCACGGGAAAGACACAGGTA |
| COX-2 | F: TGAGTACCGCAAACGCTTCTC |
|  | R: TGGACGAGGTTTTTCCACCAG |
| TNF-α | F: CTTCTCATTCCTGCTTGTG |
|  | R: ACTTGGTGGTTTGCTACG |
| iNOS | F: AGCAACTACTGCTGGTGGTG |
|  | R: TCTTCAGAGTCTGCCCATTG |
| Csf 2 | F: TCAAAGAAGCCCTGAACCTCC |
|  | R: GTGAAATTGCCCCGTAGACC |
| Mybpc2 | F: AAACTGGATGTCCGAGTGCC |
|  | R: CTGGTGTCCGGGTTCTGATG |
| Heg1 | F: CCACCTCTAACCCCAGTCAAC |
|  | R: GGCACACACATCGATACCCA |
| Gsta2 | F: CCAGGACTCTCACTAGACCGT |
|  | R: TTCAAACTCCACCCCTGCTG |
| Nr4a1 | F: GAGTTCGGCAAGCCTACCAT |
|  | R: GTGTACCCGTCCATGAAGGTG |
| Stfa2 | F: CCTGCCCAGCAATGACTGAA |
|  | R: AAGCAGTGGTCTGACCTTGT |
| Spib | F: TGGGGGCCTTGACTCTACA |
|  | R: GGGCTATAGGTGGAGGGGTT |
| Trdc | F: CTGTTTGCCAAGACCATTGCCATC |
|  | R: TGAAGCACTGAGAAGTTGGAAGCC |
| GAPDH | F: CACTCACGGCAAATTCAACGGCA |
|  | R: GACTCCACGACATACTCAGCAC |
